# Adaptive Quantum Dot Biointerfaces for Precision Wound Repair

**DOI:** 10.3390/nano16120774

**Published:** 2026-06-19

**Authors:** Hossein Omidian, Kwadwo Amanor Mfoafo, Luigi X. Cubeddu

**Affiliations:** 1 Department of Pharmaceutical Sciences, Barry and Judy Silverman College of Pharmacy, Nova Southeastern University, Fort Lauderdale, FL 33328, USA; 2 School of Pharmacy, William Carey University, Biloxi, MS 39532, USA; kmfoafo@wmcarey.edu

**Keywords:** quantum dots, precision wound repair, theranostic dressings, nanozyme biointerfaces, diabetic wounds, antibiofilm

## Abstract

Impaired wound healing arises from interacting biological and material challenges, including persistent infection, biofilm formation, oxidative stress, unresolved inflammation, impaired angiogenesis, defective epithelialization, hemorrhage, and insufficient real-time assessment of wound status. Quantum dot (QD) and nanodot nanosystems have emerged as a versatile class of bioactive wound interfaces capable of addressing these barriers through functions that extend beyond passive coverage. This review synthesizes the design rationale, material composition, validation strategies, functional outcomes, mechanistic interpretation, and translational relevance of QD-enabled platforms for precision wound repair. Across the reviewed literature, carbon dots, graphene QDs, black phosphorus QDs, metal and metal oxide QDs, transition-metal nanodots, and hybrid nanocomposites were incorporated into hydrogels, films, sponges, nanofibers, microneedles, scaffolds, membranes, sprays, and injectable matrices. Their major precision-enabling attributes include localized antimicrobial and antibiofilm activity, redox-adaptive behavior, photothermal and photodynamic activation, inflammatory and macrophage modulation, hemostasis, controlled therapeutic delivery, angiogenic and epithelial support, and fluorescence-based monitoring. The strongest conceptual advance is the transition from static wound dressings toward adaptive biointerfaces that can sense, respond to, or compensate for local wound state abnormalities. Nevertheless, the field remains largely preclinical, with important gaps in long-term safety, standardized characterization, clinically predictive models, manufacturing reproducibility, regulatory alignment, and human validation. Future progress will depend on rationally simplified multifunctional platforms, rigorous comparative testing, wound state-specific evaluation frameworks, and translation-oriented safety and usability studies. QD nanosystems therefore represent a promising foundation for precision wound repair, provided that their multifunctionality is matched by equally rigorous evidence of safety, reproducibility, and clinical relevance.

## 1. Introduction

Wound repair is a coordinated biological process that can be derailed by infection, biofilm persistence, oxidative injury, excessive inflammation, impaired vascularization, defective epithelial restoration, bleeding, and inadequate monitoring of wound progression. These barriers are especially pronounced in diabetic ulcers, chronic wounds, infected burns, traumatic injuries, and refractory tissue defects, where a single therapeutic mechanism is rarely sufficient. The reviewed literature positions QD and nanodot nanosystems as a response to this complexity: rather than functioning as inert dressing additives, they are designed as active wound biointerfaces capable of aligning material function with local pathology, including infection, oxidative stress, inflammation, vascular impairment, epithelial repair, hemostasis, and wound state monitoring [1,2,3,4,5,6,7,8].

The material basis for this precision-oriented strategy is broad. Carbon dots, carbon QDs, graphene QDs, doped carbon dots, black phosphorus QDs, metal and metal oxide QDs, metallic nanodots, metal–chalcogenide nanodots, single-atom nanozyme systems, and hybrid QD composites have been engineered for wound-relevant optical, catalytic, antimicrobial, redox, and delivery functions [9,10,11,12,13,14,15,16,17,18,19]. These nanoscale components have been integrated into hydrogels, sponges, films, electrospun fibers, nanofibrous scaffolds, bacterial cellulose matrices, microneedles, membranes, powders, and injectable or sprayable systems, enabling close contact with the wound bed while providing hydration, structural support, controlled release, optical traceability, and stimulus responsiveness [1,4,7,8,10,19,20,21,22,23,24,25,26,27,28,29,30].

Infection control represents one of the most mature functional domains of QD-enabled wound repair. Multiple systems have been developed against wound-relevant pathogens, including *Staphylococcus aureus*, methicillin-resistant *Staphylococcus aureus* (MRSA), *Escherichia coli*, *Pseudomonas aeruginosa*, *Klebsiella pneumoniae*, *Acinetobacter baumannii*, fungal species, mixed infections, and biofilm-associated wound models [8,12,31,32,33,34,35,36,37,38,39]. Mechanistically, these platforms may disrupt bacterial membranes, alter permeability, induce cytoplasmic leakage, damage bacterial macromolecules, interfere with metabolism, release antimicrobial ions, or activate photothermal, photodynamic, photocatalytic, chemodynamic, or nanozyme-mediated bacterial killing [18,19,28,31,36,38,39,40,41,42,43,44]. The precision value of these systems lies not only in antimicrobial potency, but also in the possibility of localizing antibacterial action to infected, acidic, oxidative, light-exposed, near-infrared (NIR)-responsive, or biofilm-rich wound microenvironments [9,17,18,19,28,31,41,43,45,46,47].

Equally important is the capacity of selected QD nanosystems to regulate the host wound microenvironment. Chronic and diabetic wounds are often characterized by excessive reactive oxygen species (ROS), persistent inflammatory signaling, macrophage dysregulation, and poor angiogenic repair. Redox-active carbon dots, nanozyme-like platforms, pH-responsive bimetallic systems, thermoresponsive carbon dot gels, black phosphorus QD hydrogels, and bioactive ion-releasing nanodots have been used to scavenge or generate ROS according to therapeutic context, suppress inflammatory mediators, promote macrophage transition toward pro-reparative phenotypes, and enhance vascular or epithelial repair [7,9,12,17,19,28,29,41,48,49,50,51,52,53]. This dual capacity—to sterilize hostile wound environments while protecting or restoring host tissue—defines one of the most compelling mechanistic arguments for QD-enabled precision repair.

A further distinguishing feature is theranostic integration. Fluorescent carbon dots, graphene QDs, porous silicon hybrids, pH-responsive carbon QDs, Au/Ag and Au/Zn nanodots, and fluorescence-readable hydrogels have been configured for bioimaging, wound pH monitoring, ratiometric fluorescence, dressing-state assessment, therapeutic depletion signaling, and dressing change guidance [1,4,6,15,54,55,56,57,58,59]. These functions are highly relevant to chronic and diabetic wounds, where repeated dressing removal may disturb fragile tissue and where wound progression may be difficult to infer from macroscopic appearance alone. By coupling therapeutic activity with optical or microenvironmental feedback, QD platforms offer a conceptual bridge between advanced wound dressings and responsive wound-management systems.

The functional evidence further supports this direction. Reported outcomes include high antibacterial and antibiofilm activity, favorable cytocompatibility and hemocompatibility in selected platforms, ROS and cytokine reduction, macrophage polarization, enhanced angiogenesis, epithelialization, collagen deposition, extracellular matrix remodeling, hemostatic activity, and accelerated closure across infected, diabetic, burn, traumatic, and full-thickness wound models [9,17,22,23,33,39,48,50,51,60,61,62,63,64,65,66,67,68,69]. Preclinical validation has included cell viability and migration assays, pathogen-specific antimicrobial testing, biofilm and polymicrobial models, responsive release studies, optical monitoring assays, hemostatic models, and animal wound models with histological evaluation [1,4,6,8,15,17,19,21,23,28,30,32,35,38,39,47,50,54,55,59,61,66,70,71,72,73,74,75]. Taken together, these studies establish QD nanosystems as multifunctional candidates for precision wound repair, while also underscoring the need for stricter translational validation.

In the preparation of this review, a literature search was conducted using the Web of Science database to identify recent original research articles on quantum dot and nanodot nanosystems for wound repair. Search terms were combined using Boolean operators and included “quantum dot*” OR “carbon dot*” OR “graphene quantum dot*” OR “black phosphorus quantum dot*” OR “nanodot*” AND “wound healing” OR “wound repair” OR “diabetic wound” OR “infected wound” OR “burn wound” OR “biofilm” OR “wound dressing.” Eligible studies were original research articles that reported wound-relevant quantum dot or nanodot design, fabrication, characterization, biological validation, antimicrobial or antibiofilm activity, redox or inflammatory modulation, controlled release, theranostic monitoring, hemostasis, or regenerative outcomes. Reviews, editorials, conference abstracts, patents, non-original scholarly outputs, studies unrelated to wound repair, and studies not involving quantum dot or nanodot materials were excluded.

## 2. Biomedical Aim and Nanomaterial Design Rationale

### 2.1. Clinical Rationale for Precision-Oriented Quantum Dot Wound Platforms

Wound repair is often delayed by multiple interdependent barriers, including microbial contamination, biofilm formation, excessive inflammation, oxidative stress, impaired vascularization, uncontrolled bleeding, delayed epithelial restoration, and, in diabetic or chronic wounds, a persistently dysregulated tissue microenvironment. Across the reviewed studies, QD and related nanodot systems were developed to address these barriers through active therapeutic, diagnostic, and regenerative functions rather than passive wound coverage alone. In this section, “QD nanosystems” refers broadly to wound-care platforms based on carbon dots, graphene QDs, metal and metal oxide QDs, black phosphorus QDs, transition-metal nanodots, and hybrid QD composites. The term “nanodot” is retained where the cited studies describe ultrasmall dot-like catalytic, metallic, or inorganic systems rather than conventional semiconductor QDs. These materials were incorporated into hydrogels, films, sponges, membranes, electrospun scaffolds, microneedles, injectable matrices, powders, sprays, and creams for infected wounds, diabetic ulcers, burn wounds, full-thickness skin defects, hemorrhagic injury, and chronic non-healing wounds [1,5,7,8,9,13,16,19,20,27,28,29,33,49,65,72].

**Figure 1:**
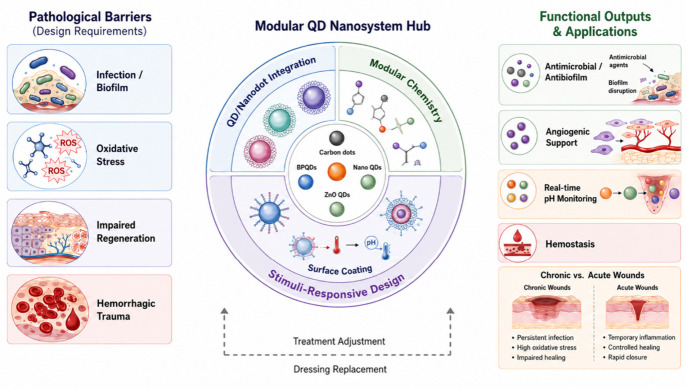
**Precision design logic of quantum dot nanosystems in wound repair.** The figure illustrates how pathological barriers—including infection and biofilm formation, oxidative stress, impaired regeneration, and hemorrhagic trauma—define design requirements for modular quantum dot and nanodot wound platforms. The central nanosystem hub integrates quantum dot/nanodot components, modular chemistry, surface coating, and stimuli-responsive design to generate functional outputs such as antimicrobial and antibiofilm activity, angiogenic support, real-time pH monitoring, hemostasis, and wound-type-specific treatment adjustment or dressing replacement (*prepared with the assistance of ChatGPT Plus (GPT 5.5) and Gemini’s NotebookLM Pro 3.5*).

Within this manuscript, the precision rationale of QD nanosystems lies in their capacity to align material function with the local wound problem. The reviewed platforms used QD properties to combine antibacterial activity, oxidative stress regulation, inflammation control, angiogenic support, bioactive delivery, hemostasis, scaffold-based tissue support, fluorescence-based monitoring, and light-activated therapy within adaptable wound-care constructs [2,3,4,5,6,7,39,66,69]. This rationale is particularly relevant because wound repair is not governed by a single biological deficit; different wound types require coordinated control of infection, immune response, vascular regeneration, extracellular matrix restoration, and epithelial closure. Figure 1 summarizes this precision-oriented design logic by linking pathological wound barriers with modular QD nanosystem functions and intended wound repair outputs.

### 2.2. Antimicrobial and Infection-Responsive Design

A major biomedical aim was the development of QD- and nanodot-based systems for infected and infection-prone wounds, including wounds complicated by multidrug-resistant organisms, MRSA, mixed bacterial populations, fungal involvement, and biofilm formation. Carbon dots, graphene QDs, silver- or copper-containing QD composites, metal oxide QDs, metal–chalcogenide nanodots, and photoactive nanodots were used as wound-contact or wound-applied materials intended to reduce microbial burden while preserving conditions compatible with tissue repair [8,11,12,18,31,32,33,34,35,36,38,39,43,45,51,76,77,78,79,80]. The shared design logic is that QD nanosystems can function as active antimicrobial interfaces, especially in resistant, polymicrobial, fungal, or biofilm-associated wound settings that require more than passive coverage.

Several platforms extended this rationale by making antimicrobial activity externally triggered or microenvironment-responsive. Photodynamic, photothermal, photocatalytic, visible-light-responsive, NIR-responsive, and second near-infrared window (NIR-II)-activated QD systems were designed to localize antimicrobial action at the wound site while supporting subsequent repair processes [9,14,17,25,37,39,43,45,46,81]. Other nanozyme-like or redox-active QD and nanodot systems addressed infection and oxidative imbalance together through design features associated with ROS generation, scavenging, or redox modulation. This reflects a precision-oriented strategy in which antibacterial action and wound microenvironment regulation are treated as linked therapeutic objectives rather than separate interventions [11,12,18,19,41,47,82,83].

### 2.3. Diabetic, Chronic, and Refractory Wound Microenvironment Regulation

Diabetic, chronic, and refractory wounds formed a central application area because they involve overlapping barriers to repair, including persistent inflammation, oxidative stress, infection susceptibility, impaired angiogenesis, metabolic imbalance, and delayed reepithelialization. QD-based hydrogels, smart dressings, injectable systems, electrospun membranes, self-contracting hydrogels, microneedle or delivery platforms, and theranostic composites were designed to intervene in these combined deficits [1,6,7,17,23,28,47,48,50,51,55,84,85,86]. The design rationale in this group is inherently multitarget: the same platform may be configured to suppress infection, scavenge excessive ROS, modulate inflammation, support vascularization, regulate the diabetic wound microenvironment, or provide mechanical and structural cues for tissue restoration.

This emphasis aligns closely with precision wound repair because chronic and diabetic wounds require intervention matched to local pathological conditions. Some systems were designed to respond to acidic, oxidative, inflammatory, infection-associated, or light-triggered wound states, whereas others combined therapeutic delivery with microenvironment regulation or fluorescence-based monitoring [1,4,6,7,15,28,41,47,55,59]. These studies frame QD nanosystems as adaptive wound-care materials whose biomedical value derives from coordinating treatment with the local wound environment.

### 2.4. Theranostic Monitoring and Wound State Feedback

A distinctive contribution of QD nanosystems is their incorporation into smart or theranostic wound platforms. Fluorescent carbon dots, carbon QDs, conjugated polymer nanodots, graphene QD-decorated porous silicon hybrids, Au/Zn and Au/Ag nanodots, pH-sensitive carbon dot composites, and fluorescence-readable hydrogels were developed to support wound state visualization, bioimaging, pH monitoring, dressing activity assessment, or dressing change guidance [1,4,6,15,49,54,55,56,57,58,59,71]. These applications extend the role of QDs beyond therapy by positioning them as components of wound-management systems that can report changes in the local wound environment.

The theranostic rationale is especially relevant for chronic and diabetic wounds, where wound progression can be difficult to assess and where unnecessary dressing removal may disturb fragile tissue. Within the reviewed studies, fluorescence, pH responsiveness, visual signaling, self-monitoring, and microenvironment-triggered release provide a material basis for more informed wound management without implying clinical validation beyond the reported evidence. These systems support the concept that QD nanosystems can combine therapeutic action with monitoring functions, strengthening their alignment with precision wound repair [1,4,6,15,55,58,59].

### 2.5. Delivery-Enabled and Regenerative Wound Matrices

Beyond antimicrobial and diagnostic functions, many QD nanosystems were designed as delivery-enabled or structurally supportive wound platforms. Carbon dot- and QD-containing hydrogels, films, nanofibers, delivery systems, and peptide- or bioactive-loaded matrices were used to support delivery of growth factors, insulin, epidermal growth factor, nitric oxide donors, curcumin, doxorubicin, gentamicin, antimicrobial peptides, and plant-derived compounds [1,7,10,29,40,45,46,74,87,88,89,90,91]. In these systems, QDs functioned as part of broader material strategies intended to improve topical delivery, sustained release, sequential release, wound-responsive treatment, or localized therapeutic bioavailability.

Other studies focused on hemostasis, burn repair, and structural tissue regeneration. QD- or carbon dot-containing sponges, hemostatic fibers, chitosan/alginate matrices, silk- or cellulose-based scaffolds, living hydrogels, nitric oxide donor systems, and nanocomposite membranes were designed to support bleeding control, wound coverage, moisture balance, tissue compatibility, or regenerative repair [20,21,50,65,66,69,74,92,93,94,95]. These applications broaden the design rationale from molecular therapy to wound-material engineering, where the nanosystem contributes to the physical, biological, and functional requirements of a regenerative dressing.

### 2.6. Bioinspired and Sustainable Quantum Dot Strategies

A substantial subset of studies used natural, herbal, food-derived, marine, agricultural-waste, insect-derived, bacterial, plant-derived, or other biomass-related sources to generate carbon dots or related QD/nanodot materials for wound repair. These platforms were derived from sources such as medicinal plants, ginger, onion peel, *Plantago major*, green tea, *Salvia miltiorrhiza*, *Eucommia ulmoides*, marigold, Tulsi, *Scutellaria baicalensis*, corn stalks, marine algae, bacterial exopolysaccharides, silkworm cocoons, beetroot, insect biomass, and other bio-derived or herbal medicine-inspired precursors [3,13,38,42,48,49,52,54,66,69,70,71,73,83,96,97,98,99,100]. Their biomedical rationale is not limited to natural origin; rather, these materials were designed to integrate wound-relevant properties such as fluorescence, antibacterial action, antioxidant capacity, anti-inflammatory activity, hemostasis, biocompatibility, or regenerative support.

This design theme also links QD development with sustainable and bioinspired material strategies. The reviewed summaries support their promise as wound-relevant materials, but claims regarding clinical superiority or broad translational readiness should remain cautious unless supported by later outcome-focused evidence.

### 2.7. Integrated Design Logic for Precision Wound Repair

Taken together, the reviewed studies establish QD nanosystems as versatile platforms for precision wound repair because they can integrate therapy, sensing, responsiveness, delivery, and regenerative support within adaptable material formats. Their biomedical aim is not confined to accelerating closure; rather, these systems are designed to intervene in the major barriers that determine wound outcome, including infection, antimicrobial resistance, oxidative stress, inflammation, angiogenic insufficiency, bleeding, burn injury, diabetic wound pathology, and inadequate monitoring. The most coherent design rationale emerging from the literature is therefore the use of QD nanosystems as multifunctional wound interfaces: materials capable of responding to wound conditions, delivering or activating therapy locally, supporting tissue regeneration, and, where applicable, providing optical or diagnostic feedback to guide wound management [2,3,5,6,7,8,16,17,50,66,69,72,86]. Table 1 summarizes the main design-rationale patterns connecting wound repair problems with QD/nanodot nanosystem architectures.

## 3. Nanomaterial Composition, Fabrication, and Physicochemical Characterization

### 3.1. Material Design Logic for Precision Wound Repair

Quantum dot nanosystems for precision wound repair are defined by the integration of four material-level design elements: nanoscale composition, surface or dopant engineering, incorporation into a wound-compatible matrix, and physicochemical validation of stimulus-responsive or traceable behavior. In this section, “quantum dots” refers broadly to nanoscale semiconductor, carbon-based, graphene-based, or hybrid fluorescent/catalytic dot systems described in the included studies, whereas “nanodots” is retained for ultrasmall metallic, oxide, chalcogenide, or catalytic dot-like structures. This distinction is important because these systems differ in core chemistry, optical behavior, catalytic activity, surface charge, and matrix compatibility, yet they converge around a shared design logic: the nanoscale dot component provides optical, catalytic, photothermal, pH-responsive, or release-modulating behavior, while the dressing matrix provides hydration, mechanical support, retention, conformability, or structural organization at the wound interface.

**Figure 2:**
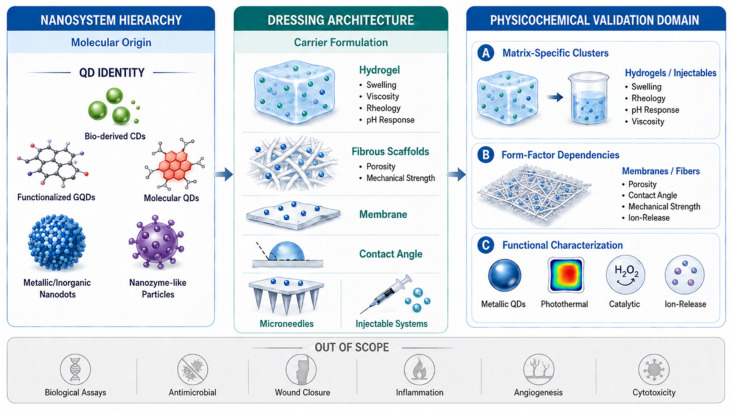
**Material design framework for quantum dot nanosystems in precision wound repair.** The schematic summarizes the section’s organizing logic: nanosystem identity and molecular origin determine QD or nanodot composition; carrier formulation translates these nanosystems into hydrogels, fibrous scaffolds, membranes, microneedles, or injectable systems; and physicochemical validation confirms matrix-specific, form-factor-dependent, and functional properties such as swelling, viscosity, rheology, pH response, porosity, contact angle, mechanical strength, photothermal behavior, catalytic activity, and ion release. Biological assays, antimicrobial testing, wound closure, inflammation, angiogenesis, and cytotoxicity are shown as outside the scope of this material-composition and characterization section (*prepared with the assistance of ChatGPT Plus (GPT 5.5) and Gemini’s NotebookLM Pro 3.5*).

Across the reviewed studies, these design elements were most often realized through carbon dots, carbon quantum dots, graphene quantum dots, doped carbon dots, semiconductor quantum dots, metallic nanodots, metal–chalcogenide nanodots, single-atom nanozymes, and metal–organic-framework-supported nanoplatforms. This section therefore focuses on what was made, how it was fabricated, and how material-level properties were confirmed. Microenvironmental responsiveness is discussed here only as a physicochemical property, such as pH-dependent fluorescence, pH-triggered decomposition, controlled release, photothermal conversion, or externally activated optical behavior, rather than as a biological outcome. Figure 2 below shows a schematic summary of the material design framework for quantum dot nanosystems in precision wound repair.

### 3.2. Composition and Surface Engineering of Quantum Dot Nanosystems

Carbon dots and carbon quantum dots (CQDs) represented one of the most frequently reported classes of wound-relevant QD materials. They were synthesized from a wide range of biomass, herbal, pharmaceutical, and biological precursors, including *Plantago major*, onion peel, bromelain, *Eucommia ulmoides*, green tea, *Salvia miltiorrhiza*, *Laminaria japonica*, ginger, beetroot, marigold flowers, Tulsi leaves, silkworm cocoon, curcumin, metformin/dopamine, corn stalks, *Scutellaria*-derived components, and tetramethylpyrazine [38,48,49,52,54,66,69,70,71,73,88,91,96,97,99,100,101]. This precursor diversity was paired with compositional tuning strategies intended to alter fluorescence, charge, dispersibility, catalytic behavior, or compatibility with polymeric carriers. Reported modifications included nitrogen, sulfur, phosphorus, iron, selenium, zinc, copper, and lanthanum incorporation, as well as amino, carboxyl, quaternary-ammonium-containing, and peptide- or ligand-associated surface functionalities [7,39,42,47,54,58,59,68,82,102,103,104].

The material landscape extended beyond CDs to include graphene QDs, black phosphorus QDs, ZnO QDs, WS_2_ QDs, Cu_2_MoS_4_ nanodots, CuS nanodots, Ag nanodots, Au/Ag nanodots, Au/Zn nanodots, vanadium oxide nanodots, copper peroxide nanodots, and gold–silver–carbon QD hybrids [9,10,11,13,14,15,16,17,18,19,26,27,45,55,60,80,89]. These inorganic and hybrid nanosystems broadened the formulation space toward photothermal, photodynamic, catalytic, pH-responsive, and fluorescence-monitoring designs. Several studies further combined QDs with metal or framework architectures, including graphene QD-coated silver nanoparticles, ceria oxide–molybdenum disulfide/CD hydrogels, AuAg-CD alginate hydrogels, and zeolitic imidazolate framework (ZIF)-Cu/C-dot metal–organic frameworks constructed from Cu single-atom nanozyme CD ligands and Zn^2+^ nodes [6,16,80,105]. Single-atom CDs with CuO_4_ or CuN_4_ coordination environments and upconversion nanocomposites co-loaded with kanamycin-derived carbon nanodots illustrate a related design direction in which QDs were embedded within more complex catalytic or optically activated nanoplatforms [12,46].

### 3.3. Matrix Integration and Dressing Architecture

The translational relevance of these nanosystems depended strongly on their incorporation into dressing architectures. Hydrogel platforms were among the most common matrix formats and included sodium alginate, chitosan, gelatin, gelatin methacryloyl (GelMA), silk fibroin, polyvinyl alcohol (PVA), carboxymethyl chitosan, oxidized sodium alginate/branched polyethyleneimine, *N*-isopropylacrylamide-based networks, bacterial cellulose, peptide-containing hydrogels, and protease- or thermoresponsive systems [4,5,8,23,25,26,29,30,39,45,47,48,68,91,94,96]. In these systems, the hydrogel served as the hydrated structural carrier, while the QD component contributed material features such as fluorescence, pH response, photothermal conversion, nanozyme-like activity, antimicrobial activity, or controlled release.

Other architectures addressed different formulation requirements. Sponge systems based on sodium alginate/PVA/CDs, chitosan/alginate/CDs, or chitosan/PVA/CDs were fabricated using electrostatic interaction, lyophilization, or freeze-drying, with emphasis on porous morphology, water absorption, hydrophilicity, and flexibility [20,65,95]. Fibrous and scaffold-based systems included spray-printed and electrospun CQD/silica/silk fibroin dressings, Mg-CQD-loaded polycaprolactone (PCL) scaffolds, PCL/collagen/ZnO QD scaffolds, chitosan-CQD-TiO_2_-graphene oxide mats, gelatin/chitosan/PCL nanoscaffolds containing Ag-CQDs, glycidyl methacrylate (GMA) hydrogel membranes containing lignin CDs combined with solution-blow-spun polylactic acid (PLA) mats, and solution-blow-spun PLA mats containing lignin CDs and curcumin [10,21,22,106,107,108,109]. Additional formats included solvent-cast chitosan/PVA QD membranes, PVA nanodot composite films, graphene QD dry powders, and selenium-doped CQD (Se-CQD)/astilbin sequential-release systems for diabetic wound repair [7,27,57,102,110,111]. Together, these formats show that QD wound materials were not limited to dispersed nanoparticles, but were engineered into carriers capable of providing structural integrity, fluid handling, tunable release, and material stability.

### 3.4. Fabrication Routes and Physicochemical Confirmation

Fabrication approaches were selected according to both nanodot chemistry and final dressing architecture. Hydrothermal synthesis was a frequently used route for plant-, drug-, and metal-modified CDs and QDs, while microwave-assisted methods were used for onion-derived, bromelain-derived, ginger-derived, beetroot-derived, insect-derived, and Cu_2_MoS_4_ nanodot systems [18,22,49,54,66,70,71,73,85,88,98,99,112]. Other preparation strategies included one-pot pyrolysis, solvothermal synthesis, ethanol-thermal synthesis, green chemical reduction, coprecipitation, redox polymerization, free-radical polymerization, calcium-ion crosslinking, Schiff-base crosslinking, ultraviolet (UV) crosslinking, solvent casting, electrospinning, spray printing, solution blow spinning, three-dimensional (3D) bioprinting, and top-down synthesis from graphite [11,21,25,26,27,31,33,38,60,87,90,94,104,106,108]. These routes enabled the integration of nanoscale optical or catalytic components into mechanically stable, hydrated, porous, injectable, fibrous, membrane-based, or powder-based architectures suitable for wound-interface application.

Physicochemical characterization provided the evidence linking fabrication to material function. Fourier transform infrared (FTIR) spectroscopy and X-ray diffraction (XRD) were widely used to confirm functional groups, bonding patterns, crystalline phases, or graphitic features, while scanning electron microscopy (SEM), transmission electron microscopy (TEM), high-resolution transmission electron microscopy (HR-TEM), field-emission scanning electron microscopy (FESEM), and atomic force microscopy (AFM) verified nanoscale morphology, particle size, porosity, membrane structure, and fiber architecture [20,27,33,66,70,73,78,85,90,99,106]. Ultraviolet–visible (UV–Vis), photoluminescence, and fluorescence spectroscopy were central for confirming optical behavior, including blue, blue-green, red, pH-responsive, and ratiometric fluorescence [4,6,49,54,58,59,88,90,97,113]. X-ray photoelectron spectroscopy (XPS), Raman spectroscopy, energy-dispersive X-ray spectroscopy (EDX/EDS), and synchrotron small-angle X-ray scattering were used in selected systems to support elemental composition, bonding state, graphitic structure, or polymer–nanodot interactions [4,13,27,66,78,97,98,114]. Dressing-level validation included swelling analysis, water uptake, water vapor transmission, rheology, contact-angle testing, degradation behavior, mechanical testing, tensile or compressive strength, self-healing, conductivity, photothermal conversion, and controlled-release assessment [4,8,10,29,31,32,47,48,68,87,88,94,106,108,115].

### 3.5. Quantitative Material Properties and Precision-Enabling Features

The reported physicochemical values support the classification of these systems as nanoscale, optically active, and formulation-dependent materials. Many reported nanodot cores fell within the sub-10 nm range, including 0.5–5 nm for beetroot CQDs, 1.02 nm for *Glenea cantor* CDs, 2.3 nm for ginger-derived CDs and AAB-CDs, 2.84 nm for EUO-NAC-CDs, approximately 3 nm for Fe-CDs, 3.6 ± 0.9 nm for strontium-doped berberine CQDs, approximately 4 nm for Cu_2_MoS_4_ nanodots, 5 ± 2 nm for chitosan-eugenol CDs, 5–7 nm for exopolysaccharide (EPS)-mediated silver nanodots (AgNDs), approximately 9.28 nm for nitrogen- and sulfur-doped graphene QDs, and 9.47 ± 0.02 nm for bromelain CQDs [13,18,49,60,71,73,82,88,98,103,114,116]. Surface and optical values further distinguished these systems: reported zeta potentials included +8.5 mV for LP-CDs, −10.03 mV for bromelain CQDs, −15.8 mV for marigold CDs, −33.7 mV for EPS-mediated AgNDs, and +30.9 mV for AAB-CDs, while quantum yields included 24.7% for LP-CDs, 91.7% for bromelain CQDs, and 12.9% ± 0.42% for silkworm cocoon-derived CDs [13,38,66,88,97,103]. Optical parameters included 450/520 nm excitation/emission for onion-derived carbon nanodots, 365 nm fluorescence for bromelain CQDs, 610 nm maximum emission for red-emitting CDs at pH 7.4, 380 nm maximum photoluminescence for marigold CDs, and pH-responsive fluorescence across pH 5.0–9.5 for functionalized CQDs [54,58,88,97,113].

At the matrix level, quantitative properties clarified how QDs were translated into wound dressing formats. Bromelain CQD hydrogels exhibited pH values of 3.6–4.4 and viscosities of 11.7–20.6 P, while p(SPA)/pectin@Ag systems released 91.6% cefazolin and p(SPA)/pectin@QD systems released 81.2% doxorubicin over approximately 300 h [87,88]. GA/WS-CQD hydrogels sustained release for up to 60 h with cumulative release exceeding 90%, and *Salvia miltiorrhiza* CD-loaded gels released 67.12% over 72 h with an 83.99% contraction ratio at 37 °C [40,48]. Structural and formulation values included bacterial nanocellulose composites approximately 2 mm thick with 120 nm microfibrils, KCD/propolis 3D-printed scaffolds with 54.01 µm pores and compressive strength of 1450.5 ± 25.4 kPa, Cu_2_ZnSnSe_4_ QD loading of 3.3 wt%, Cu_2_ZnSnS_4_ QD loadings of 0–3.3% *w*/*w*, ZCBCH hydrogel shear strength of 2.39 kPa and moisture vapor transmission rate of 26.3 g·m^−2^·day^−1^, and bacterial cellulose hydrogels containing approximately 11.7 wt% graphene QDs with 13% actual release [8,32,68,89,110,111]. Stimulus-responsive material parameters included 57.3% photothermal conversion efficiency for mesoporous silica-modified Ag_2_S QD hydrogels, 40.65% for CD-C nanocomposites, sol–gel transition at approximately 34 °C for PTPH-SCD hydrogels, NIR-induced heating to 55 °C in black phosphorus QD-based hydrogels, and CuO_2_ nanodot decomposition under mildly acidic pH 5.5–5.6 conditions [9,17,19,31,47,115]. These values demonstrate that precision in this section is grounded in measurable material attributes: size, surface charge, optical response, matrix loading, release behavior, mechanical performance, and stimulus responsiveness. Table 2 below summarizes the various composition-fabrication-characterization patterns of quantum dot nanosystems for precision wound repair.

## 4. Experimental Validation Models and Assessment Methods

**Figure 3:**
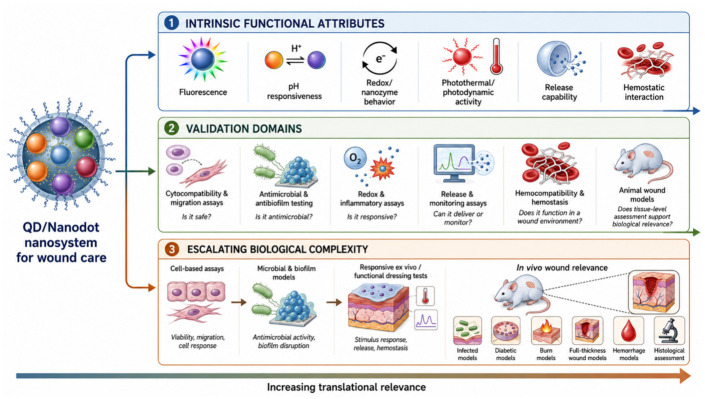
**Precision-oriented validation framework for quantum dot/nanodot nanosystems in wound repair.** The figure illustrates how intrinsic functional attributes of quantum dot/nanodot systems—including fluorescence, pH responsiveness, redox or nanozyme behavior, photothermal or photodynamic activity, release capability, and hemostatic interaction—map onto validation domains such as cytocompatibility and migration assays, antimicrobial and antibiofilm testing, redox and inflammation assays, release and monitoring assays, hemocompatibility and hemostasis, and animal wound models. The lower panel shows escalating biological complexity from cell-based assays and microbial or biofilm models to responsive ex vivo or functional dressing tests and in vivo wound models, including infected, diabetic, burn, full-thickness, hemorrhage, and histological assessment contexts (*prepared with the assistance of ChatGPT Plus (GPT 5.5) and Gemini’s NotebookLM Pro 3.5*).

Experimental validation of quantum dot and nanodot nanosystems for precision wound repair requires more than confirmation of general biocompatibility or antimicrobial activity. Because these systems are often designed to interact with dynamic wound microenvironments, their assessment has typically combined cell-based compatibility testing, pathogen-specific antimicrobial models, responsive release studies, optical or pH-sensitive monitoring, hemostatic evaluation, and wound-specific animal models. Figure 3 summarizes this precision-oriented validation logic by linking intrinsic nanosystem functions to validation domains and increasing biological complexity. This section is restricted to the experimental models, assays, biological systems, technical testing conditions, and tissue-level assessment methods used to evaluate these nanosystems. Synthesis routes, material characterization results, therapeutic conclusions, mechanistic interpretation, and efficacy outcomes are excluded, except where quantitative values define validation conditions such as pH, wavelength, release duration, exposure time, model type, dose, or biological context.

### 4.1. Cellular Compatibility and Wound-Relevant Biological Assessment

The first validation layer establishes whether QD and nanodot systems can be assessed safely in wound-relevant biological environments. Cytotoxicity, cytocompatibility, proliferation, and viability assays were widely applied using 3-(4,5-dimethylthiazol-2-yl)-2,5-diphenyltetrazolium bromide (MTT), MTT/fluorescein diacetate (FDA), and related methods across fibroblast, epithelial, endothelial, cancer-cell, and stem-cell models. These included National Institutes of Health 3T3 (NIH 3T3) and L929 fibroblasts, HaCaT cells, human foreskin fibroblast 1 (HFF-1) cells, neonatal or human dermal fibroblasts, human umbilical vein endothelial cells (HUVECs), HeLa cells, HCT 116 cells, MCF-7 cells, 3T3-L1 fibroblasts, human fibroblasts, and human amniotic membrane-derived stem cells [21,23,56,64,66,74,85,88,90,101,103,106,109,113,114]. This cellular breadth is important for precision wound repair because the same nanosystem may need to contact fibroblasts, endothelial cells, epithelial cells, immune-responsive cells, blood components, or stem-cell-based regenerative platforms depending on the wound context.

Migration and closure-related assessments provided a second cellular layer, linking biocompatibility to repair-associated cell behavior. Scratch assays, Transwell assays, fibroblast migration tests, epithelial-cell migration models, HUVEC migration assays, and mesenchymal stem cell tracking approaches were used to evaluate wound-relevant movement and repopulation processes [8,21,22,56,62,66,73,74,77,85,88,97,98,101]. Blood-contact compatibility was assessed through hemolysis, hemocompatibility, erythrocyte-preservation assays, blood-contact analysis, and related clotting or coagulation-compatible models, particularly for sponges, hydrogels, injectable systems, and dressings intended for direct wound or hemorrhage contact [20,23,30,62,65,66,68,69,76,92,95,116]. Oxidative-stress and inflammatory-response assessments included 2,2-diphenyl-1-picrylhydrazyl (DPPH) antioxidant assays, ROS-scavenging tests, macrophage polarization studies, quantitative reverse transcription polymerase chain reaction (qRT-PCR), Western blotting, transcriptomic analysis, ribonucleic acid (RNA) sequencing, cytokine-marker evaluation, and angiogenesis-marker assessment [29,49,51,52,53,59,62,70,71,85,118]. Together, these assays move validation beyond generic cytotoxicity by testing whether the nanosystems can be examined under wound-relevant cellular, inflammatory, oxidative, and blood-contact conditions.

### 4.2. Antimicrobial, Biofilm, and Pathogen-Specific Validation

A second major validation axis concerned infection control, with testing designed to capture both planktonic organisms and more complex wound-infection contexts. Antimicrobial assessment included minimum inhibitory concentration (MIC) and minimum bactericidal concentration testing, optical-density assays, disk- and agar-diffusion methods, micro-broth dilution, plate-counting assays, bacterial adhesion tests, microbial penetration assays, resistance-evolution analysis, and antibiofilm models [18,21,32,33,34,36,39,63,67,70,78,90,102,109,114]. The pathogen panels reflected clinically relevant wound-infection contexts and included *Staphylococcus aureus*, MRSA, *Escherichia coli*, *Pseudomonas aeruginosa*, *Klebsiella pneumoniae*, *Acinetobacter baumannii*, *Staphylococcus epidermidis*, *Streptococcus mutans*, *Streptococcus agalactiae*, *Candida albicans*, *Candida tropicalis*, *Aspergillus brasiliensis*, and mixed bacterial populations [8,17,27,33,35,36,45,80,99,109,114,117].

Biofilm-oriented validation added a more stringent infection model for chronic, infected, and diabetic wound contexts. Studies incorporated monomicrobial and polymicrobial biofilms, MRSA biofilms, *Candida albicans*–*S. aureus* cocultures, cellulose-supported bacterial adhesion systems, microbial penetration tests, and bacterial cellulose or hydrogel-based infection models [8,35,39,40,47,78,79,109]. This distinction is important for experimental framing: planktonic antibacterial testing establishes baseline susceptibility, whereas biofilm, polymicrobial, fungal, and penetration models provide a closer approximation of infection barriers encountered in complex wounds.

### 4.3. Responsive Delivery, Optical Monitoring, and Stimulus-Activated Testing

QD and nanodot systems are particularly relevant to precision wound repair when they are validated not only as passive materials but also as responsive delivery, sensing, or stimulus-activated platforms. Release and delivery studies evaluated antibiotics, chemotherapeutics, curcumin, gentamicin, nitric oxide (NO), silver ions, extracellular vesicles, astilbin, photosensitizer CDs, and other therapeutic or nanodot payloads [7,24,25,30,31,40,74,87,89,90,91]. Representative delivery conditions included approximately 300 h release monitoring for cefazolin and doxorubicin systems, 60 h sustained-release evaluation, 72 h thermoresponsive release assessment, 48 h gentamicin release in phosphate-buffered saline (PBS) at pH 7.4, NO-release-rate testing in cellular or glutathione peroxidase-containing environments, sequential-release Se-CQD/astilbin systems, and hydrogel-removal models based on Cu^2+^-alginate competitive complexation or calcium alginate mineralization/dissolution [7,40,48,74,87,90,119,120]. These assessments clarify how precision may be operationalized experimentally through timed, sustained, sequential, or wound-environment-responsive release.

Optical and diagnostic validation provided a complementary assessment layer. Fluorescence bioimaging, fluorescence microscopy, FRET, ratiometric fluorescence monitoring, pH-responsive fluorescence, smartphone-based UV imaging, fluorescence-intensity tracking, and wound pH monitoring were used to evaluate diagnostic or dressing-state readout functions [1,4,6,15,41,54,55,58,59,88]. Reported technical conditions included 365 nm fluorescence observation, 450 nm excitation and 520 nm emission monitoring, pH-responsive testing across pH 5.0–9.5, and UV-light-assisted smartphone imaging [6,54,58,88]. Photoactivated and microenvironment-responsive validation included photodynamic, photothermal, photocatalytic, and nanozyme assays using 450 nm visible light, white light-emitting diode (LED) irradiation, 808 nm NIR irradiation, 1064 nm laser irradiation, NIR-II irradiation, UV–Vis–NIR exposure, ROS-generation assays, glutathione-depletion assays, acidic wound-relevant pH conditions, mildly acidic pH 5.5–5.6, and wound-temperature monitoring under irradiation [9,14,17,18,19,26,31,37,39,43,46,47,81]. This group of methods is central to the manuscript’s precision theme because it shows how nanosystems were evaluated under defined optical, acidic, oxidative, or infection-associated conditions.

### 4.4. Hemostatic, Histological, and In Vivo Wound Models

For nanosystems intended to contact bleeding wounds or traumatic injuries, validation extended to hemostatic and blood-interaction models. Hemolysis, hemocompatibility, erythrocyte-preservation assays, blood-clotting index, coagulation testing, platelet-aggregation-related assays, rat liver injury, rat tail transection, mouse coagulation-disorder models, rat femoral or hepatic hemorrhage models, and rat tail, liver, or leg injury models were used to evaluate blood-contact performance, coagulation-related activity, and hemostatic suitability [30,50,65,66,68,69,92,95]. These models distinguish wound closure from bleeding control, which is a separate validation requirement for hemostatic dressings and trauma-oriented nanosystems.

In vivo wound models provided the most translationally oriented validation layer by testing nanosystems across wound severity, disease state, infection status, and tissue-repair context. Reported models included full-thickness skin wounds, full-thickness cutaneous wounds, skin defect models, diabetic rat and mouse wounds, type 1 diabetic Wistar rat wounds, diabetic ulcers, infected diabetic wounds, MRSA-infected diabetic wounds, chronic wounds, acute wounds, burn wounds, deep partial-thickness burns, third-degree burns, combined radiation-wound injury, splint-fixed infection models, rodent incisional, excision, and full-thickness wound models, lipopolysaccharide (LPS)-stimulated wounds, zebrafish wound models, *Drosophila* excision wound models, and *Galleria mellonella* burn wound infection models [8,17,19,23,28,30,32,35,38,39,47,50,54,61,64,67,72,73,74,75,101,117]. Histological and tissue-level assessment included hematoxylin and eosin (H&E) staining, Masson’s trichrome staining, microscopy, immunohistochemistry, epithelialization, collagen deposition, angiogenesis, vascularization, epidermal regeneration, hair follicle regeneration, matrix deposition, and inflammatory or oxidative-stress marker analysis [10,19,51,52,61,71,72,74,75,107]. Review-based references contributed methodological mapping rather than primary experimental evidence by classifying CD wound-healing models, CD hydrogel validation frameworks, chronic-wound applications, diabetic-wound contexts, burn-related applications, and stage-specific wound-assessment strategies [2,3,5].

Taken together, the validation landscape shows that QD and nanodot systems for precision wound repair are most clearly assessed when biological safety, pathogen relevance, microenvironment responsiveness, optical readout, controlled delivery, blood-contact suitability, and wound-specific in vivo models are integrated within a coherent experimental framework. This structure helps distinguish general wound-dressing evaluation from precision-oriented validation, in which the testing model is selected according to wound state, infection complexity, inflammatory or oxidative microenvironment, bleeding risk, and the intended diagnostic or therapeutic function of the nanosystem. At the same time, the predominance of cell-based assays, small-animal models, and model-specific wound contexts means that these validation strategies should be interpreted as preclinical assessment frameworks rather than direct clinical surrogates. Table 3 summarizes the major validation domains and their corresponding precision wound repair functions.

## 5. Functional Performance, Safety, and Quantitative Outcomes

**Figure 4:**
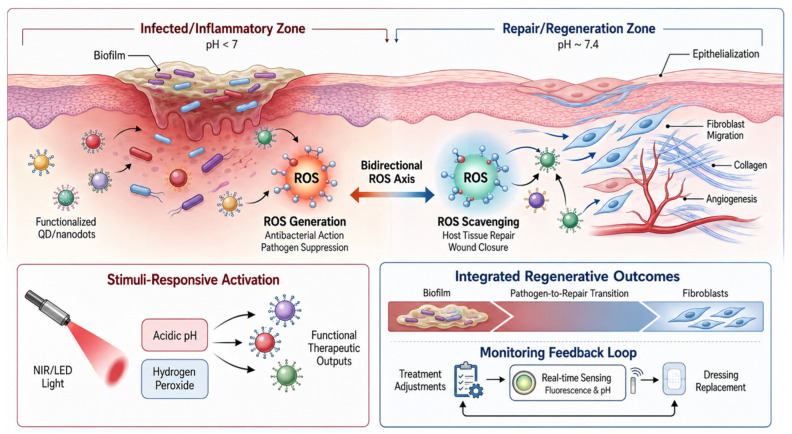
**Bidirectional ROS regulation and microenvironment-responsive QD/nanodot function in precision wound repair.** The schematic illustrates how functionalized QD/nanodot systems operate across infected/inflammatory and repair/regeneration wound zones. In acidic, biofilm-rich wound regions, stimuli such as low pH, hydrogen peroxide, and NIR/LED light can activate antibacterial responses through ROS generation, photothermal activity, and pathogen suppression. In the repair zone, ROS scavenging supports host tissue repair, wound closure, fibroblast migration, collagen deposition, angiogenesis, and epithelialization. The figure also highlights the monitoring feedback loop enabled by fluorescence- and pH-responsive sensing, which can inform treatment adjustment and dressing replacement (*prepared with the assistance of ChatGPT Plus (GPT 5.5) and Gemini’s NotebookLM Pro 3.5*).

Quantum dot and nanodot nanosystems for wound repair have been evaluated through a broad set of functional endpoints, including antimicrobial and antibiofilm activity, cytocompatibility, hemocompatibility, systemic tolerance, redox regulation, inflammatory modulation, hemostasis, controlled therapeutic release, fluorescence-guided monitoring, and regenerative outcomes such as cell migration, angiogenesis, epithelialization, collagen or extracellular matrix deposition, and wound closure. Within the framework of precision wound repair, these outcomes indicate that QD/nanodot-based materials can function as biologically interactive wound interfaces rather than passive dressing additives. Their strongest value emerges when localized infection control, wound-microenvironment regulation, tissue regeneration, and real-time or stimulus-responsive activity are integrated within the same therapeutic system. This precision-oriented logic is summarized conceptually in Figure 4 and mapped by outcome domain in Table 4.

### 5.1. Antimicrobial and Antibiofilm Performance

Localized infection control is one of the most consistently supported functions of QD/nanodot nanosystems. The reviewed platforms showed activity against common and clinically difficult wound pathogens, including *Staphylococcus aureus*, MRSA, *Escherichia coli*, *Pseudomonas aeruginosa*, *Klebsiella pneumoniae*, *Acinetobacter baumannii*, fungal species, mixed bacterial infections, and biofilm-associated wounds [8,12,31,32,33,34,35,36,37,38,39,47]. This antimicrobial activity is central to precision wound repair because it enables therapeutic action at the wound interface, including against resistant, polymicrobial, fungal, or biofilm-protected microbial populations.

Representative quantitative outcomes demonstrate the strength of this evidence base. Carbon quantum dots@silver nanoparticles (CQDs@AgNPs) showed MIC values of 0.117 mg/mL for *E. coli*, 3.75 mg/mL for *K. pneumoniae*, *A. baumannii*, and *S. aureus*, and 15 mg/mL for *C. albicans*, with MBC values matching the MIC values [33]. Ag_2_S QD hydrogels inhibited *E. coli* by 99.7% and MRSA by 99.8% under 808 nm irradiation [31], while photoresponsive CDs eliminated 98.4% of *E. coli* and 99.2% of *S. aureus* at 75 µg/mL after 10 min of light exposure [34]. Single-atom Cu-SLCDs eliminated more than 98.6% of bacteria at 25 µg/mL [12], and CD-C nanocomposites achieved greater than 98% bacterial inhibition at 150 µg/mL while disrupting biofilms [47]. In biofilm-associated wound infection, CSP/OD/CQDs-CE hydrogels achieved 91.1% in vitro and 90.6% in vivo biofilm clearance, outperforming the corresponding 6S Ag control values of 54.8% and 64.0% [39]. Together, these findings position antibacterial and antibiofilm performance as one of the better quantified functional domains within QD-enabled wound repair.

### 5.2. Safety, Cytocompatibility, and Hemocompatibility

For QD/nanodot nanosystems to be credible wound-contact materials, antimicrobial potency must be balanced with tissue compatibility and blood safety. Across the reviewed studies, safety was assessed through cell viability, fibroblast proliferation, hemolysis, organ safety, erythrocyte preservation, renal clearance, systemic tolerance, or absence of obvious toxicity. Several representative systems maintained favorable viability profiles, including bromelain CQD hydrogels with cell viability above 80% up to 20 mg/mL [88], chitosan (CS)-CQDs-MXene scaffolds with viability above 80% and 125.58% proliferation at 72 h [62], gelatin/GQD hydrogels with more than 90% human skin fibroblast viability [63], and porous nanocomposite hydrogel dressings with 100% cell viability [64].

Hemocompatibility was supported by low hemolysis values in selected platforms, including 0.98% for CS-CQDs-MXene scaffolds [62] and 2.75% for ZCBCH hydrogels, which also showed 109.90% cell viability [68]. Other systems reported negligible systemic toxicity, no obvious side effects, no major normal-tissue damage, renal clearance, preservation of erythrocytes, or safety in more complex biological settings [9,18,19,30,40,98,121]. These findings are especially relevant to precision wound repair because they indicate that QD/nanodot systems can be designed to interact with cells, blood, and wounded tissue while maintaining compatibility with the local biological environment. At the same time, the safety evidence summarized here remains tied to the reported preclinical assessments and should be interpreted within that experimental context.

### 5.3. Redox Regulation and Inflammatory Modulation

Chronic, diabetic, infected, and burn wounds are frequently characterized by persistent oxidative stress and dysregulated inflammation. QD/nanodot nanosystems addressed these barriers through ROS scavenging, ROS-responsive behavior, antioxidant activity, cytokine reduction, macrophage polarization, and inflammatory microenvironment modulation [4,7,17,29,33,41,48,49,51,52,53,55,59,74,118]. This functional domain links QD performance directly to wound state correction rather than bacterial killing alone.

Representative quantitative outcomes show that several systems achieved measurable antioxidant and anti-inflammatory effects. CQDs@AgNPs exhibited 63.90% radical-scavenging activity [33], while *Salvia miltiorrhiza* carbon dot (SM-CD) thermoresponsive gel achieved 91% ROS scavenging and reduced tumor necrosis factor alpha (TNF-α) to 16.25 ± 2.69% and interleukin 6 (IL-6) to 10.49 ± 2.04% [48]. Ginger-derived CDs decreased TNF-α, interleukin 1 beta (IL-1β), IL-6, and nitric oxide messenger RNA (mRNA) levels by 51.6%, 81.7%, 86.0%, and 58.7%, respectively [49]. ZnO-EGCG@H reduced TNF-α by 46.9% and IL-6 by 57%, while increasing vascular endothelial growth factor (VEGF) and epidermal growth factor (EGF) expression [51]. KCD/Prop scaffolds downregulated TNF-α by 4.6-fold and IL-6 by 1.5-fold, while upregulating the epithelialization marker EGF by 3.8-fold [32]. These results support the interpretation that QD/nanodot nanosystems can contribute to precision repair by reshaping the wound microenvironment toward conditions that favor regeneration.

### 5.4. Regenerative Repair and Wound Closure

Beyond antimicrobial and anti-inflammatory effects, QD/nanodot nanosystems were evaluated for direct regenerative outcomes, including fibroblast and endothelial cell migration, angiogenesis, epithelialization, collagen deposition, extracellular matrix organization, hair follicle regeneration, and wound closure. Mg-CQD-loaded PCL scaffolds improved cell migration, neovascularization, epithelialization, and collagen deposition, with VLDM enhancements of 0.611–0.749% for CQDs and 0.802–1.19% for CQD-Mg relative to reference controls [22]. Stem-cell-laden hybrid carbon-nanodot dermal matrix hydrogels promoted cell migration, early angiogenesis, superior collagen deposition, rapid closure, complete reepithelialization, and organized dermal–epidermal junction formation after 21 days [23].

The quantitative closure data are particularly compelling. In vitro, cellulose nanofiber/fucoidan/nitrogen- and sulfur-doped GQD (CNF/FUC/N,S-GQD) hydrogels achieved 99.8% scratch-wound closure within 48 h compared with 55.3% in controls [60]. In vivo, PDA-TiO_2_@Ag patches produced 99.2% closure after 7 days [61], black phosphorus QDs@nanohydrogel (BPQDs@NH) achieved 95% closure after 12 days [9], and GQDs@Cur achieved 98 ± 1.20% closure by day 9 compared with less than 50% closure in untreated and povidone-iodine controls [67]. EGCG-BPQDs@H improved diabetic burn wound closure to 92.4% after 21 days compared with 61.1% in controls [17], while ZnO-EGCG@H reached 96.3% closure after 15 days compared with 65.4% in controls [51]. In biofilm-infected wounds, CSP/OD/CQDs-CE hydrogels achieved a 99.7% final healing ratio compared with 83.0% for Tegaderm [39]. These findings show that QD/nanodot nanosystems can influence multiple phases of wound repair, linking infection suppression and microenvironment regulation to vascularization, matrix formation, epithelial restoration, and closure.

### 5.5. Hemostasis and Trauma-Oriented Dressing Function

A subset of QD/nanodot systems extended functional performance to acute bleeding control and practical wound-dressing needs. Chitosan/alginate/CD sponges showed no obvious cytotoxicity, suitable hemocompatibility, and improved hemostatic potential, with increasing CD concentration decreasing the in vitro blood-clotting index [65]. CS/PVA/CD sponges similarly demonstrated favorable biocompatibility, nontoxicity, significant in vitro clotting ability, and high in vivo hemostatic efficiency [95].

The strongest quantitative hemostatic evidence was reported for silkworm cocoon-derived CDs, which reduced rat liver bleeding time from 152.67 ± 4.16 s to 55.33 ± 9.50 s and rat tail bleeding volume from 1.71 ± 0.16 g to 0.4 ± 0.11 g. In coagulation-disorder models, an 8 mg/kg dose reduced bleeding volume to 11.80% ± 0.39% of control [66]. Tetramethylpyrazine carbon quantum dots (TMP-CQDs) also supported hemostasis through platelet aggregation and factor VIII activation while contributing antibacterial activity in infected wounds [69]. These outcomes broaden the relevance of QD/nanodot nanosystems from chronic and infected wound repair to trauma-associated wound management, where rapid hemostasis and biological compatibility are both functionally important.

### 5.6. Controlled Release, Dressing Handling, and Local Therapeutic Delivery

Controlled release and dressing usability are important precision-oriented functions because they determine whether therapy can be sustained, localized, and coordinated with the wound microenvironment. Several QD-containing systems provided sustained antimicrobial, chemotherapeutic, or nanodot release. p(SPA)/pectin@Ag hydrogels achieved 91.6% cumulative cefazolin release over approximately 300 h, while p(SPA)/pectin@QD composites released 81.2% doxorubicin over approximately 300 h [87]. GA/WS-CQD hydrogels released WS-CQDs for up to 60 h with cumulative release above 90% [40], and CQD-crosslinked chitosan films provided controlled gentamicin release over 48 h while maintaining more than 80% fibroblast viability [90]. Other systems linked release or therapeutic activity to wound microenvironment features, including H_2_O_2_-responsive GQD-decorated porous silicon dressings, pH-responsive PECDs, dual-responsive C-dot alginate patches, sequential-release Se-CQD/astilbin systems, and bacterial cellulose/GQD hydrogels with measured GQD release [1,4,7,8,59].

Dressing handling was also addressed through hydrogel dissolution and wound-care usability. CD1/4 at 90 mg/mL dissolved Cu^2+^-alginate hydrogels within 16 min, approximately twice as fast as lysine alone; the replaced hydrogels also relieved hypoxia, reduced local inflammation, and accelerated burn wound healing [119]. Calcium alginate hydrogel dissolution systems similarly supported rapid gel–sol transition, antibacterial activity, and hypoxia relief [120]. These functions are not merely technical conveniences; within a precision wound-care framework, they connect material responsiveness to atraumatic dressing management and sustained local therapy.

### 5.7. Fluorescence Monitoring, Bioimaging, and Theranostic Feedback

A distinctive contribution of QD/nanodot nanosystems to precision wound repair is their capacity to integrate therapy with optical reporting, pH sensing, cell tracking, or dressing-state feedback. Fluorescence-enabled systems included onion-derived carbon nanodots with excitation/emission at 450/520 nm [54], red-emitting CDs with maximum emission at 610 nm [113], and functionalized CQDs with a linear pH-monitoring range of 5.0–9.5 [58]. Conjugated polymer (CP) nanodots enabled long-term mesenchymal stem cell (MSC) tracking without impairing MSC proliferation, migration, differentiation, or secretome function [56]. GQD-decorated porous silicon dressings provided H_2_O_2_-responsive drug release and a red-to-blue ratiometric fluorescence shift while enhancing proliferation, migration, and diabetic wound repair [1].

Several platforms paired monitoring with treatment. PECDs and LAMC/CD-C@M@P hydrogels combined pH-responsive fluorescence with antibacterial, antioxidant, and anti-inflammatory behavior [6,59]. Au/AgND-containing hydrogels used decreasing fluorescence intensity as a visual indicator of dressing replacement timing while also providing antibacterial, photothermal, anti-inflammatory, angiogenic, and collagen-promoting effects [15]. These systems directly support the manuscript’s precision-wound repair theme because they couple wound state assessment with therapeutic function, enabling treatment to be guided by local biological signals rather than applied as a static intervention.

## 6. Mechanistic Interpretation and Translational Relevance

### 6.1. Precision Activity Within the Wound Microenvironment

QD and nanodot nanosystems contribute to wound repair by functioning as active regulators of the wound microenvironment rather than as passive covering materials. In this section, the terms are used according to the material descriptions in the cited studies and encompass CDs, GQDs, BPQDs, metal, metal oxide, or inorganic QDs, and related nanodot platforms with wound-relevant optical, catalytic, antimicrobial, redox, hemostatic, or delivery functions. Their relevance to precision wound repair lies in the ability to match nanosystem function to specific wound state abnormalities, including bacterial infection, biofilm persistence, oxidative stress, chronic inflammation, impaired angiogenesis, delayed epithelial repair, uncontrolled bleeding, and limited visibility of wound status. Across the reviewed studies, therapeutic effects were most consistently linked to integrated control of infection, redox imbalance, immune dysregulation, vascularization, epithelial restoration, hemostasis, localized therapeutic release, and optical or pH-based wound monitoring. This multifunctionality is particularly important because diabetic wounds, infected wounds, burns, traumatic wounds, and chronic ulcers do not share a single pathology; rather, each requires a different combination of antibacterial, immunomodulatory, regenerative, hemostatic, and monitoring functions [1,4,6,7,17,29,39,41,65,66,69,92,95].

**Figure 5:**
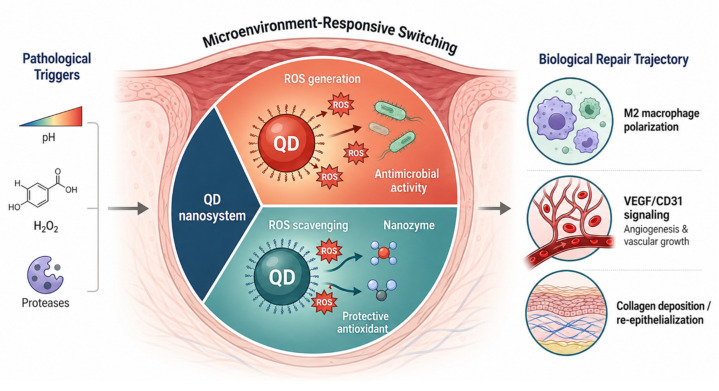
**Quantum dot nanosystems as microenvironment-responsive platforms for precision wound repair.** The figure illustrates how pathological triggers within the wound microenvironment, including pH variation, H_2_O_2_, and protease activity, can activate or guide quantum dot nanosystem functions. Depending on the wound state, these systems may promote ROS generation for antimicrobial activity or ROS scavenging through nanozyme-like and protective antioxidant behavior. These microenvironment-responsive functions converge toward biological repair trajectories that include M2 macrophage polarization, VEGF/CD31-associated angiogenesis and vascular growth, collagen deposition, and re-epithelialization (*prepared with the assistance of ChatGPT Plus (GPT 5.5) and Gemini’s NotebookLM Pro 3.5*).

The precision logic emerging from these studies is not based on one dominant mechanism, but on the design of nanosystems that respond to, report, or compensate for specific wound microenvironmental barriers. Some platforms generate ROS, heat, radicals, singlet oxygen, or NO-related therapeutic signals to sterilize infected tissue or support repair, whereas others scavenge excessive ROS to protect host cells and support regeneration. Some systems deliver ions, growth factors, NO, CDs, or bioactive compounds in a sustained or stimuli-responsive manner, whereas others use intrinsic fluorescence, pH sensitivity, or FRET-associated color changes to monitor wound status or guide dressing replacement [1,4,6,15,17,19,25,31,39,55,58,59,74,87,88]. This coupling of therapeutic activity with local responsiveness or optical feedback is central to the manuscript’s focus on QD nanosystems for precision wound repair. The microenvironment-responsive logic underlying these systems is summarized conceptually in Figure 5.

### 6.2. Antibacterial and Antibiofilm Mechanisms

Infection control was one of the most recurrent mechanistic functions. CD and QD platforms suppressed bacterial growth through direct membrane interaction, electrostatic capture, increased permeability, cytoplasmic leakage, deoxyribonucleic acid (DNA) or protein damage, metabolic interference, and inhibition or disruption of biofilms [36,38,40,42,43,44]. These effects were strengthened when QDs were integrated with antimicrobial peptides, polylysine, chitosan, botanical agents, or metal-containing components, indicating that nanosystem design can combine contact-based pathogen targeting with chemical or photoactivated antibacterial effects [32,39,45,122]. For instance, antimicrobial peptide-modified copper-doped CQD hydrogels combined membrane disruption, ROS generation, and photothermal damage, clearing MRSA biofilms by 91.1% in vitro and 90.6% in vivo [39]. GA/WS-CQD hydrogels supported sustained antibacterial activity by releasing WS-CQDs for up to 60 h with cumulative release exceeding 90%, while also inhibiting bacterial biofilm formation [40]. Amino-functionalized CDs (CDs-NH_2_) reduced biofilm formation by more than 50% at concentrations below 62.5 µg/mL, illustrating how nanoscale surface chemistry can be linked to antibiofilm function [35].

Metal- or ion-associated antibacterial effects provided a second infection-control strategy. Systems incorporating or releasing Ag^+^, Zn^2+^, Cu-containing, La-doped, vanadium-associated, or other metal-based components broadened antibacterial activity and, in some cases, contributed additional pro-regenerative, redox-regulatory, or anti-inflammatory functions [11,18,19,28,31,39,79,102,105]. In this context, the translational value of QD/nanodot nanosystems lies in their ability to combine direct pathogen suppression with local material functions such as biocompatibility, sustained release, biofilm disruption, and wound-bed support. These features support their development as antibacterial biomaterials for infected wounds, MRSA-associated lesions, burns, and diabetic ulcers.

### 6.3. Redox-Adaptive Therapy, Nanozyme Activity, and Light-Triggered Sterilization

A defining feature of many QD wound systems was context-dependent redox activity. In infected environments, ROS generation, hydroxyl radical production, singlet oxygen formation, photothermal heating, photocatalytic activity, or peroxynitrite formation contributed to bacterial killing [9,17,25,31,34,43,45,46,47]. In chronic, diabetic, or inflammatory wound settings, however, excessive ROS required antioxidant or ROS-scavenging activity to protect fibroblasts, reduce inflammatory damage, and support tissue regeneration [33,48,49,53,54,55,84,96,121]. This capacity to either generate or remove ROS according to therapeutic context is one of the clearest mechanistic bases for precision wound repair.

Nanozyme systems further refined this redox control. Peroxidase-like, oxidase-like, catalase-like, superoxide dismutase (SOD)-like, and multienzyme activities enabled selective antibacterial or antioxidant effects. pH-responsive bimetallic platforms generated bactericidal ROS in acidic infected environments but switched toward catalase- and SOD-like ROS detoxification under neutral conditions [41]. Single-atom Cu-SLCDs combined oxidase-, peroxidase-, SOD-, and catalase-like activities, eliminating more than 98.6% of bacteria at 25 µg/mL while reducing ROS in normal cells [12]. CMS nanodots showed pH-dependent peroxidase-like activity below pH 5.5 [18], while Fe-CDs, VO_x_ nanodots, herbal biomass-derived nanozymes, and W-GA nanodots used radical-generation, ROS-scavenging, or enzyme-mimetic mechanisms to support antibacterial or pro-repair wound treatment [11,53,82,83].

Light-triggered platforms added spatial and temporal control. Ag_2_S QD hydrogels showed 57.3% photothermal conversion efficiency, BPQD and epigallocatechin gallate (EGCG)-BPQD hydrogels raised wound temperature to 55 °C for sterilization, EGCG-BPQDs achieved 88.6% MRSA killing, and CD-C nanocomposites showed 40.65% photothermal conversion efficiency with more than 98% bacterial inhibition at 150 µg/mL [9,17,31,47]. Other systems used visible, NIR, or NIR-II activation to trigger photodynamic, photothermal, photocatalytic, chemodynamic, or peroxynitrite-boosted antibacterial effects [14,43,45,46]. These systems are mechanistically important because therapeutic activity can be localized to the infected wound region rather than delivered uniformly.

### 6.4. Inflammation Control and Immune-State Modulation

Inflammation resolution was a major determinant of therapeutic relevance, especially in diabetic and hard-to-heal wounds. Several QD systems suppressed pro-inflammatory mediators and restored immune balance through pathways including toll-like receptor 4 (TLR4)/nuclear factor kappa B (NF-κB), NF-κB-p65, nuclear factor erythroid 2-related factor 2 (Nrf2), Wnt, p53, hypoxia-inducible factor 1 (HIF-1), and macrophage-associated stress-response signaling [49,50,52,53,79,100]. Ginger-derived CDs blocked TLR4-mediated NF-κB signaling and reduced TNF-α, IL-1β, IL-6, and NO by 51.6%, 81.7%, 86.0%, and 58.7%, respectively [49]. ZnO-EGCG hydrogels reduced TNF-α by 46.9% and IL-6 by 57%, while increasing VEGF and EGF expression, linking inflammatory regulation to diabetic wound repair [51]. Corn-stalk CDs similarly reduced early ROS, downregulated inflammation, and blocked TLR4-mediated NF-κB signaling through inhibitor of nuclear factor kappa B alpha (IκBα) dephosphorylation and inhibition of p65 nuclear translocation [52].

Macrophage regulation provided another precision mechanism. Several systems promoted M2 or pro-regenerative macrophage phenotypes, restored immune homeostasis, or accelerated the transition from inflammation toward tissue repair [24,29,48,50,55,59,118]. Thermoresponsive SM-CD gels combined 91% ROS scavenging with reduced TNF-α and IL-6 and an M2/M1 ratio of 1.69 ± 0.11, illustrating how redox control and immune modulation can operate together [48]. CurCDs@iPRF-MA restored mitochondrial homeostasis under inflammatory conditions, activated oxidative phosphorylation, eliminated ROS, regulated macrophage phenotype, and promoted vascularization through autologous growth-factor release [29]. These findings indicate that QD nanosystems can contribute to precision wound repair by addressing the immune-redox dysfunction that prevents chronic wounds from entering a regenerative phase.

### 6.5. Angiogenesis, Epithelial Repair, and Regenerative Signaling

Beyond infection and inflammation control, QD platforms promoted tissue repair through fibroblast proliferation and migration, endothelial activation, epithelial restoration, collagen deposition, ECM organization, and angiogenesis [22,23,61,74,77,96,97,112]. Several studies linked these effects to specific regenerative signals, including VEGF, CD31, EGF, bFGF, hypoxia-inducible factor 1 alpha (HIF-1α), transforming growth factor beta (TGF-β), extracellular signal-regulated kinase (ERK)/p38 mitogen-activated protein kinase (MAPK), phosphoinositide 3-kinase/protein kinase B (PI3K/AKT), extracellular signal-regulated kinase 1/2 (ERK1/2), Nrf2, Wnt, and TGF-β/p38 MAPK/Snail signaling [10,17,19,51,53,71,75,77,100,112,123].

Representative examples show how QD nanosystems can promote vascular and epithelial repair through different design routes. PF-CD hydrogels increased VEGF expression fivefold, EGCG-BPQDs upregulated CD31 nearly fourfold and bFGF nearly twofold, and ZnO-EGCG hydrogels increased VEGF 1.7-fold and EGF twofold [17,51,112]. Magnesium-loaded CQDs combined PCL scaffold support with magnesium-associated pro-angiogenic and tissue-regenerative effects [22]. CuO_2_ nanodots decomposed in mildly acidic infected chronic wounds at pH 5.5–5.6 to release Cu^2+^ and H_2_O_2_, thereby combining antibacterial action with HIF-1α- and VEGF-associated angiogenesis [19]. Other systems contributed to repair through pathway activation: Ct-CQDs promoted wound healing through ERK and p38 phosphorylation in the MAPK pathway, while CDots induced epithelial–mesenchymal transition through the TGF-β/p38 MAPK/Snail pathway to enhance epithelial migration and reepithelialization [75,123]. Together, these findings position QD nanosystems as regenerative platforms capable of guiding wound repair biology rather than simply accelerating closure.

### 6.6. Hemostasis and Early Wound Stabilization

Several QD-containing platforms addressed the earliest phase of wound management by combining hemostasis with later regenerative support. CD-containing sponges, fibers, and nanodots promoted blood absorption, physical occlusion, clot formation, platelet aggregation, intrinsic coagulation, or factor VIII activation [65,66,69,92,95]. Silkworm cocoon-derived CDs reduced rat liver bleeding time from 152.67 ± 4.16 s to 55.33 ± 9.50 s and reduced rat tail bleeding volume from 1.71 ± 0.16 g to 0.4 ± 0.11 g, supporting their relevance for post-trauma hemorrhage control [66]. Gladiolus-derived CDs combined hemostasis with ROS scavenging and macrophage regulation, achieving early closure rates of 65.2% in combined radiation-wound injury and 62.5% in diabetic wounds within 3 days [50]. These findings suggest that selected QD systems may support both rapid acute stabilization and downstream tissue repair, which is important for traumatic, hemorrhagic, and refractory wounds.

### 6.7. Responsive Delivery, Theranostic Monitoring, and Scaffold Integration

The precision potential of QD nanosystems is especially evident when treatment is coupled with local feedback or microenvironment-triggered delivery. Hydrogels, microneedles, sponges, films, nanofibers, bacterial cellulose composites, and 3D-printed matrices enabled sustained, pH-sensitive, H_2_O_2_-responsive, protease-responsive, thermoresponsive, pressure-triggered, NIR-triggered, or sequential delivery of therapeutic agents [1,4,5,7,8,20,21,32,40,48,74,87,88,90,91,107]. GQD-decorated porous silicon dressings protected EGF and insulin from matrix metalloproteinase degradation, enabled H_2_O_2_-responsive release, and generated a FRET-associated red-to-blue fluorescence transition for wound monitoring [1]. Functionalized CQDs showed pH-responsive fluorescence across pH 5.0–9.5, while Au/Ag nanodot hydrogels linked fluorescence loss to therapeutic depletion and dressing-replacement timing [15,58]. Other systems used fluorescence to monitor pH, bacterial infection, wound state, therapeutic depletion, or dressing change timing [4,6,41,55,57,59].

Scaffold integration helped translate these mechanisms into practical wound platforms by improving retention, hydration, moisture balance, cell migration support, porosity, degradability, and local therapeutic function. QDs were incorporated into hydrogels, nanofibers, sponges, films, bacterial cellulose, and 3D-printed scaffolds to combine biological activity with structural wound support [8,20,21,32,60,62,63,68,106,107,108]. Bacterial cellulose hydrogels loaded with approximately 11.7 wt% GQDs showed 13% actual GQD release and upregulated pro-angiogenic genes after 72 h [8]. Zn-CD hydrogels achieved 95.79% wound recovery, 109.90% cell viability, and 2.75% hemolysis, while gelatin/GQD hydrogels maintained more than 90% human skin fibroblast viability [63,68]. CDs also enabled on-demand dressing management through Cu^2+^-alginate or calcium alginate hydrogel dissolution, supporting pain-free dressing replacement, antibacterial action, and hypoxia relief [119,120]. These design features connect nanoscale function to clinically relevant dressing behavior. The precision design logic of these platforms is summarized in Table 5, which links wound-specific barriers to QD-enabled strategies and the representative mechanistic or translational data retained for this section.

### 6.8. Translational Relevance

Taken together, the reviewed evidence supports QD nanosystems as multifunctional candidates for precision wound repair when their design is matched to wound pathology. Their most wound-relevant and translationally promising features include antibacterial and antibiofilm activity, redox-adaptive nanozyme behavior, photothermal or photodynamic sterilization, inflammatory and macrophage regulation, angiogenic and epithelial support, hemostatic function, controlled release, fluorescence-enabled monitoring, and scaffold-based wound support [1,4,5,6,7,17,29,39,40,41,47,48,50,65,66,69,86,87,90,92]. These properties are especially relevant for diabetic wounds, chronic ulcers, infected burns, MRSA biofilms, traumatic bleeding, oxidative-stress-associated wounds, and wounds with impaired vascularization. Within the limits of the provided studies, the strongest rationale for further translation lies not in any single mechanism but in the capacity of QD nanosystems to integrate diagnostic readout, localized therapy, immune-redox regulation, vascular and epithelial regeneration, and practical dressing function within adaptable wound-care platforms.

## 7. Limitations and Future Perspectives

Despite rapid progress, the field remains predominantly preclinical. Most evidence is derived from in vitro assays, ex vivo or simplified infection models, and small-animal wound models, including full-thickness wounds, diabetic wounds, infected diabetic wounds, MRSA-infected wounds, burn wounds, splint-fixed models, zebrafish, *Drosophila*, and *Galleria mellonella* systems [17,19,23,28,30,32,35,38,39,45,50,54,61,64,72,73,74,75,79,101]. These models are valuable for mechanistic and proof-of-concept validation, but they cannot fully reproduce human wound heterogeneity, vascular compromise, immune dysfunction, microbial ecology, comorbidities, dressing adherence challenges, pain, exudate variability, or long-term recurrence. Future studies should prioritize clinically realistic wound models, larger-animal validation where justified, and prospective designs that compare QD systems against current standards of care rather than against untreated or weakly matched controls.

A second limitation is heterogeneity in material composition, synthesis, surface chemistry, matrix integration, and characterization. The reviewed platforms span biomass-derived CDs, doped CDs, GQDs, BPQDs, metal and metal oxide QDs, metal–chalcogenide nanodots, single-atom nanozymes, and hybrid composites [7,9,10,11,12,13,14,15,17,18,19,26,28,38,46,47,48,49,52,54,55,60,66,68,69,70,71,73,79,80,82,88,97,99,100,102]. Although this diversity is scientifically productive, it complicates cross-study comparison and translational prioritization. Future work should report standardized physicochemical descriptors, including size distribution, surface charge, quantum yield, optical stability, degradation profile, ion release, endotoxin status, batch reproducibility, matrix loading, release kinetics, mechanical properties, swelling behavior, sterilization stability, and storage performance.

Safety also requires deeper and more systematic analysis. Many platforms reported favorable cytocompatibility, low hemolysis, negligible organ toxicity, renal clearance, or preservation of cell viability in selected experimental contexts [9,18,19,30,40,62,63,64,68,88,98]. However, antimicrobial or photothermal potency does not itself establish translational safety. Future studies should address dose thresholds, biodistribution, local retention, systemic absorption, degradation byproducts, immune sensitization, genotoxicity where relevant, repeated dosing, chronic exposure, reproductive and developmental considerations for selected materials, and safety under clinically realistic conditions such as infected exudate, necrotic tissue, ischemia, and repeated dressing replacement. This is particularly important for inorganic, metal-containing, photothermally activated, and ion-releasing systems.

The precision-wound repair concept also requires better alignment between intended function and validation model. Antibacterial claims should be tested against clinically relevant pathogens, resistant isolates, polymicrobial communities, and biofilm architectures rather than only planktonic bacteria [8,17,18,33,35,36,39,40,45,47,78,79,80,102,117]. Redox-regulating systems should be evaluated in oxidative and inflammatory wound-like microenvironments, with appropriate host–cell protection endpoints [29,33,48,49,51,52,53,59,62,70,71,118]. Theranostic systems should demonstrate that optical readouts correspond to actionable wound states, therapeutic depletion, pH shifts, infection progression, or dressing change timing [1,4,6,15,41,54,55,58,59]. Future studies should therefore adopt function-specific validation frameworks in which each platform is tested according to the wound pathology it claims to address.

Another major challenge is balancing multifunctionality with manufacturability. The strongest platforms often combine antimicrobial activity, redox modulation, immune regulation, controlled release, optical readout, hemostasis, and scaffold support [1,4,5,7,8,17,29,39,41,47,48,50,65,66,69,86,92,95]. However, each added function may increase formulation complexity, regulatory burden, cost, and failure points. Future development should distinguish essential from accessory functions and prioritize parsimonious designs that solve defined clinical problems. For example, an infected diabetic ulcer platform may require antibiofilm activity, ROS modulation, and pH-readable feedback, whereas an acute traumatic dressing may prioritize hemostasis, antimicrobial protection, and atraumatic removal.

Theranostic systems require particularly careful translational development. Fluorescence, pH response, FRET-associated color change, and dressing-depletion signals are promising, but clinical utility depends on readability through wound fluid, blood, slough, pigmentation, dressing opacity, ambient light variability, and user interpretation [1,4,6,15,55,58,59]. Future work should integrate quantitative imaging, smartphone-compatible readouts, calibration standards, signal-stability testing, and decision thresholds linked to clinical actions. Without such validation, optical responsiveness may remain a material feature rather than a clinically meaningful monitoring tool.

Finally, future research should move from demonstration of accelerated closure toward multidimensional wound-quality endpoints. Closure percentage alone may obscure differences in collagen organization, tensile strength, vascular maturity, epithelial barrier restoration, scar quality, infection recurrence, pain, exudate control, dressing comfort, and long-term tissue function. Histology, immunohistochemistry, inflammatory and oxidative markers, angiogenesis markers, biomechanical testing, microbial burden assessment, and recurrence monitoring should be integrated with practical dressing metrics such as removability, conformability, durability, sterilization tolerance, shelf life, scalability, and cost. Such evidence will be essential for determining whether QD nanosystems can progress from elegant preclinical constructs to clinically credible precision wound-care technologies.

## 8. Conclusions

QD and nanodot nanosystems have reframed advanced wound dressings as adaptive biointerfaces capable of integrating therapy, sensing, responsiveness, and regeneration. Their major promise lies in matching material function to wound state abnormalities, including infection, biofilm persistence, oxidative stress, inflammation, impaired vascularization, delayed epithelialization, bleeding, and inadequate monitoring. Across the reviewed evidence, the most compelling platforms combine localized antimicrobial action, immune-redox regulation, controlled delivery, scaffold-based tissue support, and optical feedback within wound-compatible material architectures. Yet translation will require disciplined simplification, standardized characterization, rigorous safety testing, clinically relevant models, and direct comparison with established wound-care strategies. If these challenges are met, QD nanosystems could become a foundational technology for precision wound repair, enabling wound dressings that not only cover damaged tissue but actively interpret, regulate, and guide the healing microenvironment.

### Evidence-to-Practice Roadmap

QD and nanodot nanosystems have progressed from material innovation toward biologically interactive wound-care platforms, but their path to practice remains uneven. The current evidence supports their strongest value as adaptive wound biointerfaces that combine localized antimicrobial action, redox and inflammatory regulation, controlled delivery, regenerative support, and optical or microenvironmental feedback. However, the field remains dominated by preclinical studies, heterogeneous formulations, nonstandardized endpoints, and limited evidence for long-term safety, manufacturability, and clinical usability. Table 6 synthesizes the evidence trajectory from rationale to clinical adoption, highlighting what is sufficiently established, what remains unresolved, and which priorities should guide the next stage of development.

## Figures and Tables

**Table 1:** Biomedical aim and design rationale of quantum dot nanosystems for precision wound repair.

Biomedical Aim and Nanomaterial Design Rationale	Precision Wound Repair Problem Addressed	QD/Nanodot Nanosystem Design Pattern	Precision-Repair Rationale	Representative References
Anti-infective QD wound interfaces	Infected wounds, MRSA, multidrug-resistant bacteria, mixed microbial contamination, and biofilm-associated infection	Carbon dots (CDs), graphene QDs, metal and metal oxide QDs, and Ag-, Cu-, or Zn-containing nanocomposites incorporated into hydrogels, films, membranes, fibers, cellulose systems, sponges, and dressings	Positions QD nanosystems as active antimicrobial wound interfaces rather than passive coverings, linking infection control with tissue-compatible repair support	[8,12,18,32,39,43,76,77,78,79,80]
Photoactivated and externally responsive antimicrobial platforms	Need for localized, controllable antimicrobial action in infected or resistant wounds	Photodynamic, photothermal, photocatalytic, visible-light-, NIR-, or NIR-II-responsive QD/nanodot systems	Enables spatial and temporal activation of antibacterial therapy at the wound site, supporting a precision-treatment logic	[9,14,17,25,37,39,43,45,46,81]
Diabetic, chronic, and refractory wound microenvironment regulation	Persistent inflammation, oxidative stress, infection susceptibility, impaired angiogenesis, delayed epithelialization, and diabetic or refractory wound dysfunction	QD-enabled hydrogels, injectable systems, microneedles, electrospun membranes, self-contracting scaffolds, and smart dressings	Frames QD nanosystems as multitarget platforms designed to regulate pathological wound microenvironments rather than address a single defect	[1,6,7,17,23,28,29,47,48,50,51,55,86]
Theranostic monitoring, optical tracking, and wound state feedback	Limited ability to monitor wound pH, infection status, dressing activity, appropriate dressing change timing, or repair-associated cell/material behavior	Fluorescent, pH-responsive, visually trackable, bioimaging-capable, or dressing-state-reporting systems based on CDs, graphene QDs, porous silicon, Au/Zn nanodots, and Au/Ag nanodots	Connects QD optical properties with precision wound management by combining therapy with wound-status reporting, dressing readout, or mechanistic bioimaging/tracking	[1,4,6,15,49,54,55,56,57,58,59,71]
Delivery-enabled QD wound matrices	Need for localized, sustained, sequential, or responsive delivery of wound-care or co-delivered therapeutic agents	QD-containing hydrogels, nanofibers, microneedles, films, and peptide- or bioactive-loaded matrices carrying agents such as growth factors, insulin, epidermal growth factor, nitric oxide donors, curcumin, doxorubicin, gentamicin, astilbin, or plant-derived compounds	Shows how QD nanosystems contribute to precision delivery by improving when, where, or how therapeutic agents are presented to the wound or wound-care matrix	[1,7,10,29,40,46,74,87,88,89,90,91]
Hemostatic, burn-repair, and regenerative scaffold systems	Acute bleeding, traumatic injury, burn wounds, full-thickness defects, refractory wounds, and structural tissue-regeneration needs	QD/CD-containing sponges, polysaccharide fibers, chitosan/alginate matrices, living hydrogels, cellulose systems, nitric oxide donor CDs, and regenerative scaffolds	Expands QD design beyond antimicrobial therapy toward integrated wound stabilization, tissue support, epithelial repair, and regenerative healing	[20,50,65,66,69,72,74,75,92,93,94,95]
Bioinspired and sustainable QD design strategies	Need for biocompatible, multifunctional, and potentially more sustainable wound-care materials	Herbal, food-derived, marine, agricultural-waste, insect-derived, bacterial, and other biomass-derived CDs or QD/nanodots	Highlights a recurring material design route in which bio-derived QDs are used to combine fluorescence, antibacterial, antioxidant, anti-inflammatory, hemostatic, or regenerative functions	[3,13,38,42,52,54,66,69,70,71,73,96,97,98,99,100]

**Table 2:** Composition–fabrication–characterization patterns of quantum dot nanosystems for precision wound repair.

Nanomaterial Composition, Fabrication, and Physicochemical Characterization: Precision Design Pattern	Representative QD/Nanodot Nanosystem and Dressing Format	Fabrication or Integration Strategy	Key Physicochemical or Formulation Data	Representative References
Biomass-, herbal-, food-, and bioactive-molecule-derived CDs as the dominant QD platform	CDs/CQDs derived from plant, herbal, food, insect, marine, protein, pharmaceutical-like, or biological precursors and formulated as hydrogels, gels, sprays, sponges, or topical systems	Hydrothermal synthesis, microwave-assisted synthesis, one-pot pyrolysis, and solvothermal synthesis	Reported nanoscale sizes included 0.5–5 nm, 1.02 nm, 2.3 nm, 2.84 nm, and 9.47 ± 0.02 nm; reported quantum yields included 24.7%, 91.7%, and 12.9% ± 0.42%; optical properties included blue, blue-green, red, and pH-responsive fluorescence	[38,49,66,71,73,88,97,98,99]
Doped and surface-functionalized CDs/CQDs for tunable material behavior	Nitrogen-, sulfur-, and phosphorus-doped CDs, Fe-CDs, Se-CQDs, Zn-CDs, Cu-containing CQDs, La-doped CQDs, and amino- or carboxyl-modified CQDs in films, hydrogels, microneedles, or smart dressing systems	Heteroatom doping, metal coordination, surface amination or carboxylation, and coordination bonding	Material tuning was reflected in zeta potential, pH-responsive fluorescence, catalytic behavior, and matrix compatibility; examples included pH response across 5.0–9.5 and high positive zeta potential of 30.9 mV for AAB-CDs	[7,54,58,59,68,82,102,103,104]
Graphene QD systems for structurally integrated wound dressings	Graphene QDs (GQDs), nitrogen- and sulfur-doped GQDs, GQDs@Ag, GQDs@Cur, C-GQDs, and GQD–bacterial cellulose composites in gelatin hydrogels, dry powders, bacterial cellulose hydrogels, nanocellulose matrices, and metal-coated nanocomplexes	Hydrothermal synthesis, top-down graphite processing, solvent casting or matrix incorporation, impregnation, green synthesis, and hybrid coating	Characterization emphasized dispersion, nanoscale morphology, optical activity, loading, and release; representative data included approximately 5 nm GQDs in gelatin hydrogels, 11.7 wt% GQD loading in bacterial cellulose, and 13% actual GQD release	[8,27,60,63,67,80,89,117]
Inorganic semiconductor QDs and nanodots for stimulus-responsive material functions	Black phosphorus QDs, ZnO QDs, WS_2_ QDs, Cu_2_MoS_4_ nanodots, Ag_2_S QDs, and CuS nanodots in hydrogels, peptide hydrogels, PCL/collagen scaffolds, or nanohydrogel systems	Hydrothermal, microwave-assisted, direct in-matrix synthesis, direct incorporation, electrospinning, and hydrogel embedding	Key properties included photothermal conversion, NIR responsiveness, pH-dependent catalytic behavior, and nanoscale morphology; examples included approximately 4 nm Cu_2_MoS_4_ nanodots, 57.3% photothermal conversion for Ag_2_S QD hydrogels, and NIR heating to 55 °C in black phosphorus QD hydrogels	[9,10,14,17,18,26,31,45]
Metallic nanodots and metal–QD hybrids for optical, photothermal, and dispersion control	Au nanodots, Au/Zn nanodots, Au/Ag nanodots, AgNDs, AuAg-CDs, and GQDs@Ag in PVA films, alginate hydrogels, creams, and nanocomplexes	Codeposition, EPS-mediated green reduction, nanocluster embedding, QD coating of metal nanoparticles, and hydrogel incorporation	Representative values included approximately 2.5 nm SFT/DT-Au nanodots, 5–7 nm EPS-mediated AgNDs, and AgND surface charge of −33.7 mV; QD coating improved dispersion and stability in metal-containing hybrids	[13,15,16,55,76,80]
Nanozyme, single-atom, and metal–organic framework (MOF)-supported QD systems	ZIF-Cu/C-dots, Cu-SLCDs, CuN-CDs, VO_x_ nanodots, CuO_2_ nanodots, and CuO_2_–MgO_2_ bimetallic peroxide nanodots in MOF, sprayed, hydrogel, or fibrous membrane formats	MOF assembly, solvothermal synthesis, ethanol-thermal synthesis, coordination synthesis, and polydopamine (PDA) anchoring	Characterization centered on coordination environment, enzyme-like activity, component release, pH-triggered decomposition, and metal-ion responsiveness; CuO_2_ nanodots were designed to decompose at pH 5.5–5.6	[11,12,19,28,105]
Hydrogel-integrated QD dressings as the main translational formulation format	CD/CQD-, SCD-, CuS-, Zn-CD-, CurCD-, and CD-C-loaded hydrogels based on alginate, chitosan, GelMA, silk fibroin, gelatin, PVA, decellularized matrix, polycarbonate, and peptide-containing networks	Ionic crosslinking, Schiff-base chemistry, UV crosslinking, freeze–thaw cycling, free-radical polymerization, and in situ gelation	Hydrogel properties included swelling, viscosity, self-healing, conductivity, release, moisture vapor transmission, and photothermal conversion; examples included pH 3.6–4.4 and viscosity of 11.7–20.6 P for bromelain CQD hydrogels, 2.39 kPa shear strength and 26.3 g·m^−2^·day^−1^ moisture vapor transmission rate (MVTR) for ZCBCH, and 40.65% photothermal conversion for CD-C	[4,23,25,26,29,30,39,47,68,88,91,96,115]
Fibrous, membrane, scaffold, sponge, and powder architectures for structural control	QD-loaded PCL, PVA, chitosan, collagen, gelatin, PLA, bacterial cellulose, and nanofibrous composites	Electrospinning, spray printing, solution blow spinning, solvent casting, freeze-drying, lyophilization, and 3D printing	Material characterization emphasized porosity, hydrophilicity, water uptake, flexibility, degradation, and mechanical strength; examples included 54.01 µm scaffold pore size, 1450.5 ± 25.4 kPa compressive strength, 3.3 wt% Cu_2_ZnSnSe_4_ QD loading, and Cu_2_ZnSnS_4_ QD loadings of 0–3.3% *w*/*w*	[20,21,22,27,32,65,89,95,106,107,108,110,111,116]
Monitoring-enabled and stimulus-responsive QD systems for precision dressing design	pH-responsive CQDs, ratiometric GQD@porous silicon systems, fluorescent Au/AgND hydrogels, study-specific fluorescent CD systems, and LAMC/CD-C@M@P hydrogels	Fluorescent QD embedding, nanochannel confinement, fluorescence resonance energy transfer (FRET)-based design, pH-responsive surface chemistry, and smartphone-readable fluorescence	Readouts included pH-linear fluorescence, red-to-blue fluorescence transitions, reversible pH-dependent signals, dressing-state fluorescence decrease, and NIR-triggered heating; these properties support material-level monitoring and externally triggered activation	[1,4,6,9,15,17,58,59]

**Table 3:** Experimental Validation Models and Assessment Methods for Quantum Dot Nanosystems in Precision Wound Repair.

Experimental Validation Models and Assessment Methods	Precision Wound Repair Function Being Validated	Representative Models, Assays, and Technical Conditions	Pattern Revealed for the Manuscript	Representative References
Cellular compatibility and reparative cell behavior	Establishes whether the nanosystem is suitable for contact with wound-relevant cells and whether it can be evaluated in migration or repair-associated contexts	MTT, MTT/FDA, cytotoxicity, cytocompatibility, proliferation, scratch, Transwell, and migration assays using NIH 3T3, L929, HaCaT, HFF-1, neonatal dermal fibroblasts, HUVECs, HeLa, MCF-7, HCT 116, 3T3-L1, human fibroblasts, and stem-cell models	Validation moves beyond generic cytotoxicity when fibroblast, endothelial, epithelial, and migration assays are combined, making the testing more relevant to wound repair than general biocompatibility alone	[21,23,56,64,66,74,88,90,98,106,109,113,114]
Oxidative-stress, inflammatory, and immune-response assessment	Tests whether the model captures inflammatory and redox features of impaired wounds, especially diabetic, infected, and chronic wounds	DPPH antioxidant assays, ROS-scavenging assays, macrophage polarization assessment, qRT-PCR, Western blotting, RNA sequencing, transcriptomic analysis, cytokine-marker evaluation, and angiogenesis-marker assessment	Precision-oriented validation frequently incorporates wound microenvironment readouts, especially oxidative stress and inflammation, rather than relying only on wound-size or survival endpoints	[49,51,52,53,59,62,70,71]
Antimicrobial susceptibility and pathogen-specific testing	Determines whether the nanosystem is tested against wound-relevant pathogens and resistant organisms	MIC, minimum bactericidal concentration (MBC), optical-density assays, disk diffusion, agar diffusion, micro-broth dilution, plate counting, bacterial adhesion assays, and testing against *S. aureus*, MRSA, *E. coli*, ampicillin-resistant *E. coli*, *P. aeruginosa*, *K. pneumoniae*, *A. baumannii*, *S. epidermidis*, *S. mutans*, and *S. agalactiae*	The most relevant antimicrobial validation aligns the assay type with clinically meaningful wound pathogens, especially MRSA and Gram-negative bacteria in infected or diabetic wound contexts	[8,17,18,21,33,51,63,90,99,102,109,114,117]
Biofilm, polymicrobial, fungal, and barrier-penetration models	Assesses whether testing reflects the complexity of infected wounds rather than planktonic bacteria alone	Antibiofilm assays, monomicrobial and polymicrobial biofilms, MRSA biofilms, *Candida albicans*–*S. aureus* cocultures, microbial penetration tests, cellulose-supported bacterial adhesion models, and fungal models involving *C. albicans*, *C. tropicalis*, and *A. brasiliensis*	Biofilm, polymicrobial, fungal, and penetration models provide a higher-resolution validation layer for chronic or infected wounds, where planktonic antibacterial assays alone are insufficient	[35,39,40,47,78,79,109]
Controlled release, sequential delivery, and dressing-removal assessment	Evaluates whether QD systems function as delivery platforms, responsive reservoirs, or removable dressings	In vitro release of cefazolin, doxorubicin, curcumin, gentamicin, NO, silver ions, extracellular vesicles, astilbin, and photosensitizer CDs; representative conditions include approximately 300 h release monitoring, 60 h sustained release, 72 h thermoresponsive release, 48 h gentamicin release in PBS at pH 7.4, NO-release-rate testing, sequential-release microneedles, Cu^2+^-alginate dissolution, and calcium alginate mineralization/dissolution	This validation layer links precision repair to timed, sustained, sequential, or environment-responsive delivery, while also capturing dressing-removal strategies that reduce disruption to the wound bed	[7,8,25,30,31,40,48,74,87,90,91,119,120]
Optical imaging, pH monitoring, and theranostic readout	Tests whether QDs provide diagnostic or monitoring functions in addition to therapeutic evaluation	Fluorescence bioimaging, fluorescence microscopy, FRET, ratiometric fluorescence, pH-responsive fluorescence, smartphone-based UV imaging, dressing-state fluorescence tracking, wound pH monitoring, 365 nm fluorescence observation, 450/520 nm excitation–emission monitoring, and pH-responsive testing across pH 5.0–9.5	This is the clearest connection to precision wound repair: QDs are validated not only as materials or drugs, but also as wound state sensors and dressing-monitoring components	[1,6,15,41,54,55,58,59,88]
Photoactivated, nanozyme, and microenvironment-responsive testing	Evaluates activation under wound-relevant stimuli such as light, pH, redox state, and peroxide-rich environments	Photodynamic, photothermal, photocatalytic, and nanozyme assays using 450 nm visible light, white LED irradiation, 808 nm NIR, 1064 nm laser irradiation, NIR-II irradiation, UV–Vis–NIR exposure, ROS generation, glutathione depletion, pH < 5.5, pH 5.5–5.6, and wound-temperature monitoring under irradiation	Stimulus-based validation distinguishes precision nanosystems from passive dressings by showing how activity can be tested under defined optical, acidic, oxidative, or infection-associated conditions	[14,17,18,19,26,31,37,39,43,46,47,81]
Hemostatic and blood-contact validation	Determines suitability for bleeding wounds, traumatic injury, and direct blood exposure	Hemolysis, hemocompatibility, erythrocyte-preservation assays, blood-clotting index, coagulation testing, platelet-aggregation-related assays, rat liver injury, rat tail transection, mouse coagulation-disorder models, rat femoral/hepatic hemorrhage models, and rat tail, liver, or leg injury models	This validation category separates wound closure from bleeding control, which is essential for nanosystems intended for trauma, hemorrhage, or hemostatic dressing applications	[30,50,65,66,68,69,92,95]
In vivo wound models and histological tissue assessment	Establishes translational relevance across wound severity, disease context, infection status, and tissue repair quality	Full-thickness wounds, full-thickness cutaneous wounds, skin defect models, diabetic rat and mouse wounds, type 1 diabetic Wistar rat wounds, diabetic ulcers, infected diabetic wounds, MRSA-infected diabetic wounds, chronic wounds, acute wounds, burn wounds, deep partial-thickness burns, third-degree burns, combined radiation-wound injury, splint-fixed infection models, incisional and excision wounds, LPS-stimulated wounds, zebrafish, *Drosophila*, and *Galleria mellonella* models; H&E, Masson’s trichrome, microscopy, immunohistochemistry, epithelialization, collagen deposition, angiogenesis, vascularization, epidermal regeneration, hair follicle regeneration, and matrix deposition assessments	The most translationally informative studies pair disease-specific wound models with tissue-level evaluation, while remaining preclinical assessment systems rather than direct substitutes for clinical validation	[17,19,23,28,30,32,35,38,39,47,50,54,61,64,72,73,74,75]
Review-level methodological mapping	Places primary validation models within broader frameworks rather than serving as primary experimental evidence	Classification of CD wound-healing models, CD-hydrogel validation frameworks, chronic-wound applications, diabetic-wound contexts, burn-related applications, and stage-specific wound-assessment strategies	Review articles are best used to contextualize validation strategy and model selection, not as substitutes for primary experimental testing	[2,3,5]

**Table 4:** Functional performance, safety, and precision-relevant outcomes of quantum dot/nanodot nanosystems for wound repair.

Functional Performance, Safety, and Quantitative Outcomes	Precision Wound Repair Function	Key Outcomes, Endpoints, and Representative Quantitative Findings	Representative References
Antimicrobial and antibiofilm activity	Localized infection control against planktonic bacteria, resistant strains, and wound biofilms	Activity was reported against *S. aureus*, MRSA, *E. coli*, *P. aeruginosa*, *K. pneumoniae*, *A. baumannii*, and fungal species. Representative outcomes included MIC values of 0.117 mg/mL for *E. coli* and 3.75 mg/mL for *K. pneumoniae*, *A. baumannii*, and *S. aureus*; 99.7% *E. coli* and 99.8% MRSA inhibition under NIR irradiation; 98.4% *E. coli* and 99.2% *S. aureus* elimination after 10 min of light exposure; >98.6% bacterial elimination at 25 µg/mL; and 91.1% in vitro and 90.6% in vivo biofilm clearance	[8,12,17,31,33,34,35,39,40,47,51,67,78]
Cytocompatibility, hemocompatibility, and systemic safety	Balancing antimicrobial potency with tissue and blood compatibility	Safety endpoints included fibroblast viability, hemolysis, organ safety, erythrocyte preservation, low cytotoxicity, and systemic tolerance. Representative results included >80% viability up to 20 mg/mL, >90% human skin fibroblast viability, 100% cell viability in one hydrogel system, 0.98% hemolysis for CS-CQDs-MXene scaffolds, 2.75% hemolysis with 109.90% cell viability for ZCBCH hydrogels, and reports of negligible systemic toxicity, no major organ damage, renal clearance, or erythrocyte preservation	[9,18,19,30,40,62,63,64,68,88,98]
ROS regulation and inflammatory modulation	Correction of oxidative and inflammatory wound microenvironments, especially in chronic or diabetic wounds	QD/nanodot systems acted through ROS scavenging, ROS-responsive activity, cytokine reduction, antioxidant effects, and macrophage polarization. Representative data included 63.90% radical-scavenging activity; 91% ROS scavenging; TNF-α, IL-1β, IL-6, and NO mRNA reductions of 51.6%, 81.7%, 86.0%, and 58.7%; TNF-α and IL-6 reductions of 46.9% and 57%; and inflammatory-marker downregulation with concurrent pro-regenerative marker elevation	[7,29,32,33,48,49,51,52,53,55,59]
Regenerative repair and wound closure	Promotion of fibroblast migration, angiogenesis, epithelialization, collagen/extracellular matrix (ECM) deposition, and tissue restoration	Strong closure and repair endpoints were reported across infected, diabetic, burn, and full-thickness wounds. Representative results included 99.8% wound closure within 48 h versus 55.3% control; 99.2% closure after 7 days; 95% closure after 12 days; 98 ± 1.20% closure by day 9 versus <50% in untreated and povidone-iodine controls; 96.3% closure after 15 days versus 65.4% control; and 99.7% final healing ratio versus 83.0% for Tegaderm. Additional outcomes included increased VEGF, cluster of differentiation 31 (CD31), EGF, basic fibroblast growth factor (bFGF), collagen deposition, ECM organization, epithelialization, and hair follicle regeneration	[9,17,22,23,39,50,51,60,61,67,74,91,97]
Hemostasis and trauma-oriented wound support	Rapid bleeding control with concurrent tissue-repair compatibility	Hemostatic QD/nanodot systems reduced bleeding, supported clotting, and maintained cytocompatibility or hemocompatibility. Silkworm cocoon-derived CDs reduced rat liver bleeding time from 152.67 ± 4.16 s to 55.33 ± 9.50 s, rat tail bleeding volume from 1.71 ± 0.16 g to 0.4 ± 0.11 g, and coagulation-disorder bleeding volume to 11.80% ± 0.39% of control at 8 mg/kg. CD-containing sponges and fibers showed improved clotting ability, reduced blood-clotting index with increasing CD content, and high in vivo hemostatic efficiency	[50,65,66,69,92,95]
Controlled release and sustained local therapy	Localized therapeutic delivery with reduced reliance on passive dressing function	Release-oriented systems provided sustained antimicrobial, peptide, chemotherapeutic, nanodot, or bioactive delivery. Representative data included 91.6% cefazolin release over approximately 300 h, 81.2% doxorubicin release over approximately 300 h, >90% WS-CQD release over 60 h, controlled gentamicin release over 48 h, Ag^+^ release under NIR-responsive hydrogel conditions, and sequential or microenvironment-responsive release in diabetic wound platforms	[1,4,7,31,40,87,90]
Fluorescence monitoring, pH sensing, and theranostic feedback	Real-time wound state assessment, guided dressing replacement, and combined diagnosis–therapy	Fluorescence-enabled systems supported wound monitoring, pH sensing, cell tracking, dressing-state readout, and theranostic response. Representative outcomes included excitation/emission at 450/520 nm, red emission at 610 nm, linear pH response from 5.0 to 9.5, red-to-blue ratiometric fluorescence shift, pH-responsive fluorescence for early infection detection, long-term MSC tracking without impairing cell function, and fluorescence-based guidance for dressing replacement timing	[1,6,15,54,55,56,58,113]
Dressing handling and wound-care usability	Atraumatic dressing replacement, hypoxia relief, and practical wound-management support	Beyond direct antimicrobial and regenerative effects, selected QD/nanodot systems improved dressing-related function. CD1/4 at 90 mg/mL dissolved Cu^2+^-alginate hydrogel within 16 min, approximately twice as fast as lysine alone, while replaced hydrogels relieved hypoxia, reduced local inflammation, and accelerated burn wound healing. Calcium alginate hydrogel dissolution systems similarly supported rapid gel–sol transition, antibacterial activity, and hypoxia relief	[119,120]

**Table 5:** Precision Design Logic of Quantum Dot Nanosystems in Mechanistic Wound Repair.

Mechanistic Interpretation and Translational Relevance	Precision Wound Repair Target	QD Nanosystem Strategy	Key Mechanistic or Translational Data Retained for This Section	Representative References
Infection- and biofilm-directed antibacterial control	Resistant bacterial infection, MRSA colonization, mixed bacterial wounds, and biofilm persistence	Surface-charged or bioactive QDs disrupt bacterial membranes, capture bacteria, damage bacterial contents, inhibit biofilms, or combine with antimicrobial polymers, peptides, ions, cellulose carriers, or botanical agents	CDs-NH_2_ reduced biofilm formation by >50% below 62.5 µg/mL; GA/WS-CQD hydrogels sustained WS-CQD release for up to 60 h with >90% cumulative release and inhibited biofilm formation; peptide-modified copper-doped CQD hydrogels cleared MRSA biofilms by 91.1% in vitro and 90.6% in vivo	[35,36,38,39,40,42,43,44,78]
Redox-adaptive and nanozyme-mediated repair	Excess oxidative stress in chronic wounds and acidic infected microenvironments requiring selective bacterial killing	QD nanozymes either generate bactericidal ROS/radicals under infected conditions or scavenge excessive ROS to protect regenerating host tissue	pH-responsive bimetallic platforms switched from peroxidase-like antibacterial ROS generation in acidic environments to catalase/SOD-like ROS detoxification at neutral pH; Cu-SLCDs eliminated >98.6% of bacteria at 25 µg/mL while reducing ROS in normal cells; CMS nanodots showed pH-dependent peroxidase-like activity below pH 5.5	[11,12,18,41,48,53,82,83]
Light-triggered precision sterilization	Localized infection, antibiotic-resistant bacteria, infected diabetic wounds, and biofilm-associated wounds	Visible, NIR, or NIR-II activation triggers photothermal, photodynamic, photocatalytic, or chemodynamic antibacterial effects with spatial control	Ag_2_S QD hydrogels showed 57.3% photothermal conversion efficiency; BPQD and EGCG-BPQD hydrogels raised wound temperature to 55 °C for sterilization; EGCG-BPQDs achieved 88.6% MRSA killing; CD-C nanocomposites showed 40.65% photothermal conversion efficiency and >98% bacterial inhibition at 150 µg/mL	[9,17,31,43,45,46,47]
Inflammation resolution and immune-state modulation	Persistent inflammation, diabetic wound dysregulation, and delayed transition from inflammation to repair	QD systems regulate inflammatory signaling, reduce cytokine expression, restore redox balance, and promote M2 or pro-regenerative macrophage polarization	Ginger-derived CDs reduced TNF-α, IL-1β, IL-6, and NO by 51.6%, 81.7%, 86.0%, and 58.7%; SM-CD thermoresponsive gels achieved 91% ROS scavenging and an M2/M1 ratio of 1.69 ± 0.11; ZnO-EGCG hydrogels reduced TNF-α by 46.9% and IL-6 by 57%	[29,48,49,50,51,52,55,59,118]
Angiogenic and regenerative pathway activation	Impaired vascularization, delayed epithelialization, and poor matrix remodeling	QD platforms deliver bioactive ions or regulate regenerative signaling to promote fibroblast migration, endothelial activation, collagen deposition, and reepithelialization	PF-CD hydrogels increased VEGF expression fivefold; EGCG-BPQDs upregulated CD31 nearly fourfold and bFGF nearly twofold; ZnO-EGCG hydrogels increased VEGF 1.7-fold and EGF twofold; CuO_2_ nanodots released Cu^2+^/H_2_O_2_ at pH 5.5–5.6 and induced HIF-1α/VEGF expression	[17,19,22,51,71,74,75,112,123]
Hemostatic stabilization with regenerative support	Acute bleeding, traumatic wounds, and early wound instability before repair	QD-containing sponges, fibers, or nanodots absorb blood, support clotting, activate coagulation-related mechanisms, and preserve a repair-supportive wound interface	Silkworm cocoon-derived CDs reduced rat liver bleeding time from 152.67 ± 4.16 s to 55.33 ± 9.50 s and rat tail bleeding volume from 1.71 ± 0.16 g to 0.4 ± 0.11 g; *Gladiolus*-derived CDs combined hemostasis with ROS scavenging and macrophage regulation in refractory wound models	[50,65,66,69,92,95]
Responsive delivery and theranostic wound monitoring	Variable wound pH, oxidative stress, protease-rich wounds, and uncertain dressing change timing	QD systems enable sustained or stimuli-responsive release and/or fluorescence, FRET, or pH-sensitive signals for wound state feedback	GQD@porous silicon dressings protected EGF and insulin from matrix metalloproteinase degradation, enabled H_2_O_2_-responsive release, and produced a FRET-associated red-to-blue monitoring signal; functionalized CQDs showed pH-responsive fluorescence across pH 5.0–9.5; Au/Ag nanodot hydrogels linked fluorescence loss to therapeutic depletion and dressing-replacement timing	[1,4,6,15,41,55,57,58,59]
Scaffold-integrated precision repair	Need for local retention, moisture balance, cell migration support, and wound-specific material function	QDs are incorporated into hydrogels, nanofibers, sponges, films, bacterial cellulose, 3D-printed matrices, or composite scaffold systems to combine therapeutic activity with structural wound support	Bacterial cellulose hydrogels loaded with approximately 11.7 wt% GQDs showed 13% actual GQD release and upregulated pro-angiogenic genes after 72 h; Zn-CD hydrogels achieved 95.79% wound recovery, 109.90% cell viability, and 2.75% hemolysis; gelatin/GQD hydrogels maintained >90% human skin fibroblast viability	[8,21,32,60,62,63,68,106,107,108]

**Table 6:** Evidence-to-Practice Roadmap for QD and Nanodot Nanosystems in Precision Wound Repair.

Translational Stage	What the Current Evidence Shows	What Remains Insufficiently Known	Evidence-to-Practice Priority	Take-Home Synthesis
Rationale: why QD/nanodot wound systems matter	Wound repair is limited by interacting barriers, including infection, biofilm persistence, oxidative stress, inflammation, impaired angiogenesis, delayed epithelialization, bleeding, and poor wound state monitoring. QD/nanodot systems are attractive because they can integrate multiple wound-relevant functions within one adaptable material interface.	It remains unclear which wound barriers most strongly justify QD/nanodot use over simpler advanced dressings in specific clinical scenarios.	Define the clinical problem first: infected diabetic ulcer, MRSA biofilm, burn wound, chronic inflammatory wound, hemorrhagic injury, or monitoring-dependent wound care.	The field should move from “multifunctional materials” toward wound state-matched precision platforms.
Mechanism: how the systems act	Representative systems support antibacterial, antibiofilm, ROS-generating, ROS-scavenging, nanozyme-like, photothermal, photodynamic, immunomodulatory, angiogenic, epithelial-supportive, hemostatic, delivery-enabled, and fluorescence-monitoring functions. The strongest mechanistic insight is context dependence: some systems sterilize infected wounds by generating ROS or heat, whereas others protect chronic wounds by reducing oxidative and inflammatory burden.	Many studies still connect material properties to biological effects indirectly. Mechanistic specificity remains incomplete for dose–response behavior, microenvironment-triggered switching, long-term redox effects, and the relative contribution of each component in complex composites.	Prioritize representative systems for deeper mechanistic dissection using causal experiments, pathway validation, component-deletion controls, and wound-relevant microenvironment models.	Mechanistic credibility will depend less on listing functions and more on proving why a given nanosystem works in a defined wound state.
Validation: from assays to translational models	Current validation includes cytocompatibility, migration assays, antimicrobial testing, biofilm models, responsive release studies, fluorescence or pH monitoring, hemostatic assays, histology, and small-animal wound models. This creates a broad preclinical evidence base.	Most models remain simplified, short term, and weakly predictive of human wounds. Rodent wounds, planktonic bacterial assays, and standard closure measurements do not fully capture human wound chronicity, comorbidities, vascular insufficiency, polymicrobial biofilms, exudate burden, pain, or recurrence.	Develop tiered validation frameworks: material safety and stability, wound-relevant in vitro assays, biofilm and immune-redox models, human skin or advanced wound models, larger-animal studies where appropriate, and eventually controlled clinical evaluation.	Validation should become pathology-specific rather than generic; the model should match the claimed precision function.
Trials: what would justify clinical testing	Preclinical data suggest potential benefit in infected wounds, diabetic wounds, burns, chronic wounds, hemorrhagic wounds, and wounds requiring monitoring or controlled release. Some systems show strong closure, antimicrobial, inflammatory, angiogenic, and hemostatic outcomes.	There is not yet enough evidence to identify which platforms are most ready for human trials, which comparator dressings should be used, or which regulatory pathway is most appropriate for combined therapeutic–diagnostic systems.	Select the simplest high-value candidates for first-in-human translation: platforms with a clear indication, reproducible synthesis, favorable safety profile, measurable added value, and clinically meaningful endpoints.	Early trials should not test “QD wound dressings” broadly; they should test one defined platform for one defined wound indication against an appropriate standard of care.
Populations: who may benefit most	The strongest rationale applies to wounds with combined pathological barriers: diabetic ulcers, chronic non-healing wounds, infected burns, MRSA or biofilm-associated wounds, oxidative-stress-dominant wounds, vascularly impaired wounds, and traumatic bleeding wounds.	Patient stratification is not yet established. It is unclear which patients need antimicrobial, redox-regulating, theranostic, hemostatic, or regenerative QD functions, and which could be managed with simpler dressings.	Link platform selection to wound phenotype, microbial burden, inflammatory/redox status, vascular compromise, exudate level, depth, and need for monitoring or atraumatic dressing change.	Precision use will require matching the nanosystem to the wound phenotype, not applying multifunctional dressings universally.
Outcomes: what should be measured	Studies commonly report wound closure, bacterial reduction, biofilm clearance, cell viability, hemolysis, ROS or cytokine changes, angiogenic markers, epithelialization, collagen deposition, fluorescence response, release behavior, and hemostasis.	Closure percentage alone is insufficient. Long-term scar quality, tensile strength, recurrence, reinfection, pain, exudate control, patient comfort, dressing change frequency, optical-readout usability, and cost-effectiveness are rarely established.	Standardize outcome sets that include biological repair quality, infection control, safety, usability, and patient-centered endpoints. For theranostic systems, define whether the optical signal changes clinical decisions.	The field should shift from “faster closure” to clinically meaningful healing quality and decision-guiding performance.
Clinical practice: how these systems could be used	QD/nanodot platforms could eventually serve as active wound interfaces that combine local treatment, wound state responsiveness, sustained delivery, infection control, regenerative support, and monitoring feedback.	Practical implementation remains uncertain, including sterilization, shelf life, storage, dosing, frequency of replacement, compatibility with wound exudate, interpretation of optical signals, clinician workflow, and regulatory classification.	Design platforms for real wound-care use: easy application, safe removal, stable signal readout, predictable dosing, compatibility with standard dressings, and clear clinical instructions.	Clinical adoption will depend as much on usability and reliability as on biological activity.
Safety and toxicity: what must be resolved before practice	Several systems report favorable cytocompatibility, hemocompatibility, organ safety, renal clearance, or low systemic toxicity in preclinical settings. Carbon-based and bio-derived systems may offer attractive safety advantages, while metal-containing systems can provide potent antimicrobial or catalytic functions.	Long-term biodistribution, local retention, degradation products, repeated exposure, heavy-metal-related toxicity, ion-release risks, immune sensitization, and safety under infected or chronic wound conditions remain insufficiently resolved.	Establish material-specific safety packages, especially for heavy-metal, metal oxide, photothermal, and ion-releasing systems. Mitigation should include surface passivation, protective coatings, controlled degradation, dose optimization, and lower-toxicity alternatives where feasible.	Safety cannot be inferred from short-term cytocompatibility; translational readiness requires material-specific toxicology.
Manufacturing and scalability: what determines feasibility	The field has demonstrated many synthesis and integration routes, including hydrothermal, microwave-assisted, solvothermal, polymer-crosslinking, electrospinning, solvent-casting, printing, sponge formation, and hydrogel incorporation strategies.	Batch reproducibility, scale-up, quality control, sterilization tolerance, storage stability, cost, regulatory consistency, and reproducible matrix loading remain underdeveloped, especially for complex multifunctional composites.	Favor simplified, reproducible formulations with measurable critical quality attributes, scalable synthesis, stable optical or catalytic function, and manufacturable dressing architecture.	The most translatable platform may not be the most multifunctional one, but the one that is reproducible, safe, stable, and clinically useful.
Future gaps: what should guide the field	The evidence supports QD/nanodot nanosystems as promising adaptive biointerfaces for precision wound repair, particularly where infection, redox imbalance, inflammation, poor vascularization, and monitoring needs overlap.	The main gaps are not conceptual but translational: standardization, safety, clinical relevance, manufacturability, and proof that QD-enabled functions improve decisions or outcomes beyond existing care.	Build an evidence pipeline from rational material design to wound-phenotype-specific validation, clinically meaningful endpoints, safety-by-design, manufacturable formulations, and carefully selected clinical indications.	The next stage should convert multifunctionality into disciplined, indication-specific translational value.

## Data Availability

No new data were created or analyzed in this study. Data sharing is not applicable to this article.

## Abbreviations

The following abbreviations are used in this manuscript: **3D**—three-dimensional; **AFM**—atomic force microscopy; **AgND**—silver nanodot; **AgNP**—silver nanoparticle; **bFGF**—basic fibroblast growth factor; **BPQD**—black phosphorus quantum dot; **CD**—carbon dot; **CD31**—cluster of differentiation 31; **CDs-NH_2_**—amino-functionalized carbon dots; **CNF/FUC/N,S-GQD**—cellulose nanofiber/fucoidan/nitrogen- and sulfur-doped graphene quantum dot; **CP**—conjugated polymer; **CQD**—carbon quantum dot; **CS**—chitosan; **DNA**—deoxyribonucleic acid; **DPPH**—2,2-diphenyl-1-picrylhydrazyl; **ECM**—extracellular matrix; **EDX/EDS**—energy-dispersive X-ray spectroscopy; **EGCG**—epigallocatechin gallate; **EGF**—epidermal growth factor; **EPS**—exopolysaccharide; **ERK**—extracellular signal-regulated kinase; **ERK1/2**—extracellular signal-regulated kinase 1/2; **FDA**—fluorescein diacetate; **FESEM**—field-emission scanning electron microscopy; **FRET**—fluorescence resonance energy transfer; **FTIR**—Fourier transform infrared; **GelMA**—gelatin methacryloyl; **GMA**—glycidyl methacrylate; **GQD**—graphene quantum dot; **H&E**—hematoxylin and eosin; **HFF-1**—human foreskin fibroblast 1; **HIF-1**—hypoxia-inducible factor 1; **HIF-1α**—hypoxia-inducible factor 1 alpha; **HR-TEM**—high-resolution transmission electron microscopy; **HUVEC**—human umbilical vein endothelial cell; **IκBα**—inhibitor of nuclear factor kappa B alpha; **IL-1β**—interleukin 1 beta; **IL-6**—interleukin 6; **LED**—light-emitting diode; **LPS**—lipopolysaccharide; **MAPK**—mitogen-activated protein kinase; **MBC**—minimum bactericidal concentration; **MIC**—minimum inhibitory concentration; **MOF**—metal–organic framework; **mRNA**—messenger RNA; **MRSA**—methicillin-resistant *Staphylococcus aureus*; **MSC**—mesenchymal stem cell; **MTT**—3-(4,5-dimethylthiazol-2-yl)-2,5-diphenyltetrazolium bromide; **MVTR**—moisture vapor transmission rate; **NF-κB**—nuclear factor kappa B; **NH**—nanohydrogel; **NIH**—National Institutes of Health; **NIR**—near-infrared; **NIR-II**—second near-infrared window; **NO**—nitric oxide; **Nrf2**—nuclear factor erythroid 2-related factor 2; **PBS**—phosphate-buffered saline; **PCL**—polycaprolactone; **PDA**—polydopamine; **PI3K/AKT**—phosphoinositide 3-kinase/protein kinase B; **PLA**—polylactic acid; **PVA**—polyvinyl alcohol; **QD**—quantum dot; **qRT-PCR**—quantitative reverse transcription polymerase chain reaction; **RNA**—ribonucleic acid; **ROS**—reactive oxygen species; **Se-CQD**—selenium-doped carbon quantum dot; **SEM**—scanning electron microscopy; **SM-CD**—*Salvia miltiorrhiza* carbon dot; **SOD**—superoxide dismutase; **TEM**—transmission electron microscopy; **TGF-β**—transforming growth factor beta; **TLR4**—toll-like receptor 4; **TMP-CQD**—tetramethylpyrazine carbon quantum dot; **TNF-α**—tumor necrosis factor alpha; **UV**—ultraviolet; **UV–Vis**—ultraviolet–visible; **VEGF**—vascular endothelial growth factor; **XPS**—X-ray photoelectron spectroscopy; **XRD**—X-ray diffraction; **ZIF**—zeolitic imidazolate framework.
